# ELISA (Embedding-Linked Interactive Single-cell Agent): an interpretable hybrid generative Artificial Intelligence agent for expression-grounded discovery in single-cell genomics

**DOI:** 10.1093/bib/bbag501

**Published:** 2026-09-10

**Authors:** Omar Coser

**Affiliations:** Independent Researcher, Via Vecchie Pontare 24, 38121 Meano, Trento, Italy, Europe

**Keywords:** AI agents, single-cell genomics, AI discovery

## Abstract

Translating single-cell RNA sequencing (scRNA-seq) data into mechanistic biological hypotheses remains a critical bottleneck, as agentic AI systems lack direct access to transcriptomic representations while expression foundation models remain opaque to natural language. Here, we introduce ELISA (Embedding-Linked Interactive Single-cell Agent), an interpretable framework that unifies single-cell generative pretrained transformer expression embeddings with biomedical bidirectional encoder representations from transformers-based semantic retrieval and large-language model (LLM)-mediated interpretation for interactive single-cell discovery. An automatic query classifier routes inputs to gene marker scoring, semantic matching, or reciprocal rank fusion pipelines depending on whether the query is a gene signature, natural language concept, or mixture of both. Integrated analytical modules perform pathway activity scoring across 60+ gene sets, ligand–receptor interaction prediction using 280+ curated pairs, condition-aware comparative analysis, and cell-type proportion estimation, all operating directly on embedded data without access to the original count matrix. Benchmarked across six diverse scRNA-seq datasets spanning inflammatory lung disease, pediatric and adult cancers, organoid models, healthy tissue, and neurodevelopment, ELISA significantly outperforms CellWhisperer, a classical lexical retriever (BM25), and a random baseline in cell type retrieval (combined permutation test, $p < 2\times 10^{-5}$ for each), with particularly large gains on gene-signature queries (Cohen’s *d = 5.98* for mean reciprocal rank). ELISA replicates published biological findings (mean composite score 0.88), and generates candidate hypotheses through grounded LLM reasoning, bridging the gap between transcriptomic data exploration and biological discovery.

## Introduction

Single-cell RNA sequencing (scRNA-seq) has transformed our understanding of cellular heterogeneity by enabling genome-wide transcriptional profiling at single-cell resolution [1]. Standardized analytical pipelines support quality control, normalization, clustering, differential expression (DE), and trajectory inference [2], catalyzing the construction of comprehensive cell atlases across tissues, developmental stages, and disease contexts. However, a critical bottleneck persists: translating statistical outputs of differentially expressed gene lists, enriched pathways, and predicted ligand–receptor (LR) interactions into mechanistic biological hypotheses remains labor-intensive, context-dependent, and difficult to scale or reproduce.

Large-language models (LLMs) offer a potential solution to this problem. LLMs encode substantial biomedical knowledge and perform competitively on clinical reasoning benchmarks [3], whereas retrieval-augmented generation (RAG) improves factual accuracy by grounding outputs in external knowledge at inference time [4]. These capabilities have motivated *agentic* AI architectures that are capable of autonomous planning, tool usage, and iterative reasoning within closed-loop workflows.

Recent agentic systems span a broad range of biomedical applications (Table 1). Towards an AI Co-Scientist [5] introduces multi-agent hypothesis generation through structured debate and evolutionary refinement, though it operates over textual knowledge without interfacing with experimental data. Biomni [6] constructs a unified action space from biomedical tools and databases, enabling dynamic task orchestration including gene prioritization. GeneAgent [7] and related systems [8] extend LLM reasoning to gene-set analysis, whereas Virtual Lab [9] demonstrates collaborative multi-agent discovery. Within single-cell analysis, CellAgent [10] decomposes scRNA-seq workflows into agent-handled subtasks, AutoBA [11] generates executable pipelines from natural language, and BRAD [12] integrates LLMs with enrichment analysis for biomarker identification. In retrieval-augmented space, GeneGPT [13] provides structured access to NCBI databases, and systems for deep phenotyping [14] and biomedical data extraction [15, 16] have demonstrated the utility of RAG for factual grounding. CRISPR-GPT [17] further illustrates agentic automation for gene-editing experiment design. However, these systems are primarily responsible for curated text and structured databases and lack the capacity to operate directly on high-dimensional transcriptomic representations.

**Table 1:** Comparison of existing AI systems for biomedical and single-cell analysis.

**System**	**Expr.**	**Sem.**	**L–R /**	**Cond.**	**Interp.**	**Primary**
	**Emb.**	**Ret.**	**Pathway**	**Comp.**	**Report**	**Scope**
AI Co-Scientist [5]	–	–	–	–	✓	Hypothesis generation
Biomni [6]	–	✓	–	–	–	General biomedical
GeneAgent [7]	–	✓	–	–	–	Gene-set analysis
Virtual Lab [9]	–	–	–	–	✓	Multi-agent discovery
CellAgent [10]	–	–	–	–	–	scRNA-seq pipelines
AutoBA [11]	–	–	–	–	–	Pipeline generation
BRAD [12]	–	✓	–	–	–	Biomarker ID
GeneGPT [13]	–	✓	–	–	–	Database querying
CRISPR-GPT [17]	–	–	–	–	–	Experiment design
scGPT [18]	✓	–	–	–	–	Cell embeddings
CellWhisperer [21]	✓	✓	–	–	–	Multimodal embedding
ELISA (ours)	✓	✓	✓	✓	✓	Interactive sc discovery

Expr. Emb., uses expression-derived embeddings from foundation models; Sem. Ret., semantic retrieval over biological annotations; L–R / Pathway, LR interaction and pathway scoring from data; Cond. Comp., condition-aware comparative analysis; Interp. Report, automated interpretive report generation with LLM.

Concurrently, foundation models for single-cell biology have achieved remarkable progress in the learning of expressive latent representations from transcriptomic data. Single-cell generative pretrained transformer (scGPT) [18] employs generative pretraining over millions of single-cell transcriptomes, capturing gene–gene dependencies for cell embedding, annotation transfer, and perturbation prediction. Extensions such as scWGBS-GPT [19] and Tokensome [20] broaden learned representations to methylomics and multimodal settings. However, these expression embeddings are not designed for semantic querying; they capture transcriptional similarity in latent spaces that lack alignment with the natural language concepts that biologists use to formulate hypotheses. Notably, CellWhisperer [21] addressed part of this gap by learning joint embeddings of transcriptomes and textual annotations via contrastive training, enabling chat-based interrogation of scRNA-seq data within CELLxGENE [21]. While this establishes a compelling proof of concept for natural-language exploration, it does not incorporate built-in analytical modules for pathway scoring, interaction prediction, or condition-aware comparison.

This landscape reveals a fundamental disconnect: agentic systems and LLM-based tools excel at reasoning over text and generating interpretations but lack direct access to transcriptional data structure, while expression foundation models learn rich cellular representations that remain opaque to natural language interfaces. No existing system has unified expression-derived embeddings with semantic language representations within a single interactive framework for single-cell discovery.

**Figure 1:**
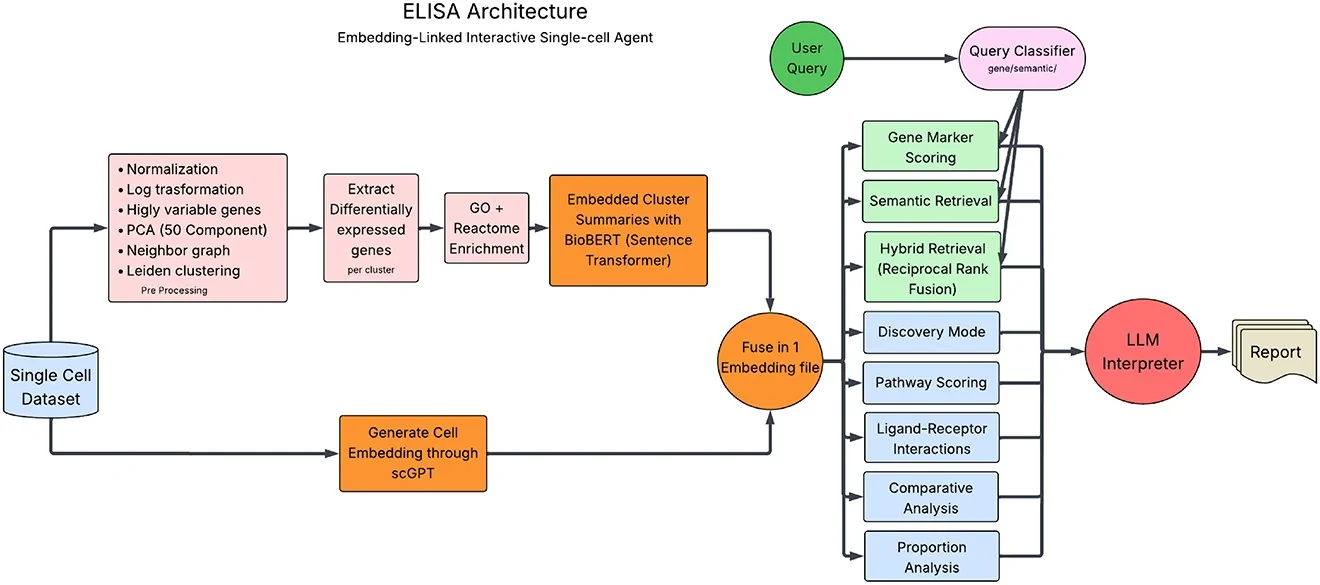
Overview of the ELISA architecture. The framework comprises three stages. In *data preparation* (left), a single-cell dataset undergoes standard preprocessing (normalization, log-transform, highly variable gene selection, PCA, neighbor graph construction, and Leiden clustering), after which per-cluster DE statistics are computed, enriched with GO and Reactome terms, and encoded into 768D semantic embeddings via BioBERT. In parallel, cell-level expression embeddings are generated through scGPT. Both representations are fused into a single serialized embedding file (.pt). In the *retrieval and analysis* stage (center), a query classifier routes user input gene signatures, natural language concepts, or mixed queries to the appropriate pipeline: gene marker scoring, semantic retrieval, or hybrid retrieval via RRF. Additional analytical modules perform pathway scoring, LR interaction prediction, comparative analysis, and proportion estimation directly on the embedded data. In the *interpretation* stage (right), all retrieval and analysis outputs are passed to a Groq-hosted LLM (LLaMA 3.1-8B) that generates grounded biological interpretations and structured reports.

**ELISA** (**E**mbedding-**L**inked **I**nteractive **S**ingle-cell **A**gent) addresses this gap by integrating scGPT expression embeddings with semantic retrieval and LLM-based biological interpretation in a unified discovery platform (Fig. 1). Rather than retraining the expression foundation models, ELISA treats scGPT cluster embeddings as an expression-side representation that is explicitly combined with BioBERT-derived semantic embeddings through an automatic hybrid routing mechanism. A query classifier detects whether the input is a gene signature, a natural language concept, or a mixture of both, and routes it to the appropriate retrieval pipeline gene marker scoring, semantic cosine similarity, or reciprocal rank fusion (RRF) of both enabling flexible navigation across the full spectrum of biological queries. Built-in analytical modules for condition-aware comparative analysis, LR interaction prediction, pathway activity scoring, and cell-type proportion analysis operate directly on the embedded data, while an LLM reasoning layer translates statistical outputs into structured biological interpretations. Critically, ELISA enforces strict separation between dataset-derived evidence and LLM-generated knowledge, enabling transparent hypothesis generation. The system produces comprehensive, publication-ready reports with Nature-style visualizations, supporting the full arc from exploratory query to structured scientific output.

We validated ELISA on six diverse scRNA-seq datasets spanning distinct tissues, disease contexts, and experimental designs. Through a systematic comparison with published findings, we demonstrate that ELISA recovers key biological signals differentially expressed genes, altered cell-type proportions, pathway activities, and cell–cell interaction networks with high fidelity. A quantitative evaluation framework comprising five complementary metrics (gene coverage, interaction recovery, pathway alignment, proportion consistency, and qualitative theme coverage) provides a principled assessment of the capacity of the system to replicate established biological conclusions. To the best of our knowledge, scGPT embeddings have not been integrated with semantic language representations in a query-conditioned retrieval framework for single-cell genomics.

In summary, this work makes the following contributions:

- Multimodal discovery agent for single-cell genomics. We introduce ELISA, an interpretable AI framework that integrates transcriptomic embeddings, semantic knowledge retrieval, and LLM reasoning to enable natural-language-driven exploration and biological discovery from scRNA seq data. Its interpretability is grounded in provenance: every hypothesis traces back to explicit retrieval scores and differential-expression values, as illustrated end-to-end in Supplementary Section S1.
- Query-adaptive hybrid retrieval architecture. ELISA employs automatic query classification and dynamic pipeline routing to combine complementary retrieval strategies—including gene marker scoring, semantic similarity search, and RRF—allowing flexible, query-conditioned navigation of complex cellular landscapes.
- Integrated biological analysis modules for expression-grounded reasoning. The system incorporates analytical components for comparative expression analysis, LR interaction scoring, pathway activity estimation, and cell-type proportion profiling, enabling automated interpretation and contextualization of discovered signals.
- Benchmarking framework for evaluating AI-assisted biological discovery. We propose a quantitative evaluation strategy that measures the ability of AI agents to recover biologically meaningful findings reported in reference studies, and apply this framework across six diverse scRNA-seq datasets.
- Empirical validation of discovery performance. Across multiple datasets and evaluation metrics, ELISA consistently recovers the majority of key biological signals reported in the corresponding studies, demonstrating its potential to support interpretable and reproducible AI-assisted discovery in single-cell genomics.

## Materials and methods

Full details of the parameters, hyperparameters, and software environment are given in Supplementary Section S13 (Supplementary Tables S6–S9) and Supplementary Section S3. Full dataset descriptions are given in Supplementary Section S10 and Table S5. A complete description of the method is given in Supplementary Section S11.

### Datasets

ELISA was validated on six publicly available scRNA-seq datasets from CZ CELLxGENE Discover (Supplementary Table S5), spanning lung (cystic fibrosis, CF) [22], adrenal tumor (neuroblastoma) [23], multi-cancer ICB [24], lung organoid [25], healthy breast tissue [26], and first-trimester brain [27]. Datasets were downloaded in AnnData format and preprocessed into a standardized embedding format. Cell type annotations from the original publications were retained without modification. Representative cell-level visualizations produced by ELISA for the CF dataset are shown in Supplementary Figs S1 and S2.

### System architecture

ELISA integrates four modules a hybrid retrieval engine, an analytical suite, a visualization toolkit, and an LLM chat interface operating on a shared serialized PyTorch embedding file per dataset. Each embedding file stores cluster identifiers, BioBERT semantic embeddings (768D), optional scGPT expression embeddings, per-cluster DE statistics, gene ontology (GO) and Reactome enrichment terms, and metadata. This cluster-level representation eliminates the need for access to the original count matrix at query time.

### Hybrid retrieval

An automatic query classifier routes each input to one of the three pipelines based on token-level heuristics. *Gene queries* (*≥ *60% gene-symbol tokens) were scored against per-cluster DE profiles using a weighted function of $|\log _{2}\mathrm{FC}|$ and expression specificity ($\mathrm{pct_{in}} - \mathrm{pct_{out}}$). *Ontology queries* are encoded with BioBERT [28] and matched to precomputed cluster description embeddings via cosine similarity, augmented by Cell Ontology name boosting (*α = 0.15*) and synonym expansion (*β = 0.10*). *Mixed queries* are resolved through RRF of both pipelines (*k = 60*). For benchmarking, an additive union strategy selects the higher-recall modality as primary and appends unique results from the secondary pipeline.

### Analytical modules

The four built-in modules operate directly on the embedded data. *LR interaction prediction* scores source–target cluster pairs using a curated database of 280+ pairs compiled from CellChat [29], CellPhoneDB [30], and NicheNet [31]. *Pathway activity scoring* quantifies 60+ curated gene sets across five categories (immune signaling, cell biology, neuroscience, metabolism, and tissue-specific). *Comparative analysis* stratifies clusters by condition metadata and identifies condition-biased gene expression. *Proportion analysis* computes per-cluster cell fractions and condition-specific fold changes. A detailed description is given in Supplementary Section S11.

### Large-language model interpretation

Retrieval and analysis outputs are interpreted by LLaMA-3.1-8B-Instant [32] via the Groq API (temperature 0.2); the API interfaces for GPT [33], Gemini [34], and Claude [35] are also integrated and ready to use. Prompts enforce strict grounding in dataset evidence, with explicit instructions to avoid hallucination and causal claims. A discovery mode generates structured outputs comprising dataset evidence, established biology, consistency analysis, and candidate hypotheses.

### Benchmarking

Retrieval was evaluated using 100 queries (50 ontologyand 50 expression) with curated expected clusters, assessed using Cluster Recall@*k* and mean reciprocal rank (MRR), the complete query sets are listed in Supplementary Section S14. ELISA was compared against CellWhisperer [21]. Analytical modules were evaluated against ground truth from source publications using interaction recovery rate, pathway alignment, proportion consistency, and gene recall. A combined permutation test (50 000 permutations) assessed overall significance across all metrics simultaneously.

## Results

### ELISA’s hybrid retrieval outperforms competing methods across datasets and query types

**Figure 2:**
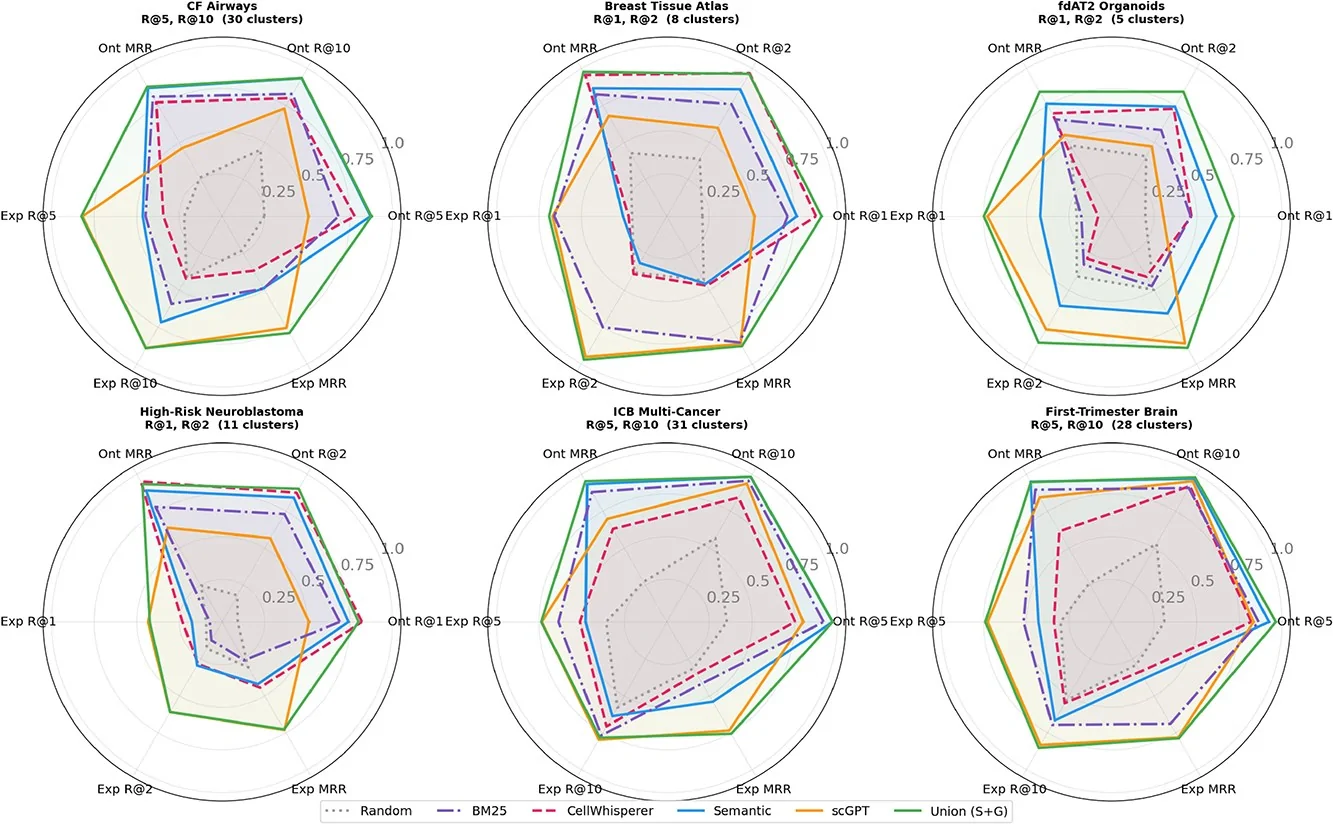
ELISA Union achieves the best retrieval performance on both query types across six datasets. Radar plots showing retrieval performance on ontology (Ont) and expression (Exp) queries for each dataset. Each plot displays six axes: Cluster Recall@k at two cutoffs and Mean Reciprocal Rank (MRR), evaluated separately on ontology and expression queries (see Supplementary Section S8 for metric definitions). The Recall@k cutoffs are adapted to each dataset's number of annotated clusters and are stated in each panel title: small-cluster datasets (Breast Atlas, 8 clusters; fdAT2 Organoids, 5; High-Risk Neuroblastoma, 11) use the stringent cutoffs R@1 and R@2, whereas large-cluster datasets (CF Airways, 30; ICB Multi-Cancer, 31; First-Trimester Brain, 28) use R@5 and R@10. Because the cutoffs differ by dataset, the corresponding axes are not directly comparable across panels; each panel is internally consistent across the six retrieval modes. Higher values (further from center) indicate better performance. Six retrieval modes are compared, as identified in the figure key: Random (dotted), BM25 (dash-dot), CellWhisperer (dashed), ELISA Semantic, ELISA scGPT, and ELISA Union. Each baseline excels on only one query type and collapses on the other: lexical (BM25) and semantic retrieval are strong on ontology but weak on expression, whereas the expression-embedding mode (scGPT) shows the reverse; Random remains near the center for large-cluster datasets and is correspondingly higher where few clusters make chance retrieval easier. ELISA Union is the only mode that consistently achieves the largest radar footprint on both ontology and expression axes, matching or exceeding every baseline on each. A combined permutation test over all 12 retrieval metrics confirms that Union significantly outperforms CellWhisperer, BM25, and a random baseline (*p* < 2 × 10^−5^ for each; see Table 2).

To evaluate the ability of ELISA to retrieve biologically relevant cell types from single-cell atlases, we benchmarked its retrieval performance against CellWhisperer [21], a state-of-the-art multimodal framework for natural-language interrogation of scRNA-seq data, as well as two additional baselines requested to contextualize the contribution of each component: a classical lexical retriever (BM25) [36] and a random ranking. For each of the six datasets (Supplementary Table S5), we designed paired sets of ontology queries (concept-level, e.g. “macrophage infiltration in CF airways”) and expression queries (gene-signature-based, e.g. “MARCO FABP4 APOC1 C1QB C1QC MSR1”), with curated expected cluster sets derived from the corresponding reference publications. We evaluated six retrieval modes: random, BM25, CellWhisperer, semantic ELISA, scGPT ELISA (gene marker scoring pipeline), and ELISA union (additive fusion of semantic and gene pipelines via adaptive routing). Performance was assessed using Cluster Recall@*k* and MRR across both query categories (Fig. 2; formal definitions of all retrieval and analytical evaluation metrics are provided in Supplementary Section S8).

Across the six datasets, the ELISA Union mode consistently achieved the highest or near-highest performance, enveloping, or matching the profile of all baselines on the radar plots (Fig. 2). Because Recall@*k* cutoffs are adapted to each dataset’s cluster count, individual Recall@*k* metrics are evaluated on the subset of datasets sharing that cutoff (*n = 3*–*6*; Supplementary Table S1), so the aggregate advantage is assessed by the combined permutation test over all 12 metrics and all six datasets rather than by any single per-cutoff metric. To quantify this advantage, we performed paired statistical tests across the six datasets for each retrieval metric (Supplementary Table S1), complemented by a combined permutation test that aggregates all 12 metrics simultaneously and is not subject to the resolution limit of the paired Wilcoxon test at *n=6*. This combined test confirmed that ELISA Union significantly outperformed CellWhisperer, BM25, and the random baseline ($p < 2\times 10^{-5}$ for each; 50 000 permutations; Table 2); the full per-metric comparisons against BM25 and against the random baseline are given in Supplementary Tables S2 and S3. The advantage over CellWhisperer was driven by large improvements on expression queries (mean *Δ *MRR = +0.41, paired *t*-test *P <.001*, Cohen’s *d* = 5.98; mean *Δ *Recall@5 = +0.29, *P =.006*, *d* = 1.57) and consistent gains on ontology queries (mean *Δ *MRR = +0.15, *P =.028*, *d* = 1.02; mean *Δ *Recall@5 = +0.08, *P =.047*, *d* = 0.84), with no dataset in which CellWhisperer held an overall advantage. The Semantic and scGPT pipelines each independently outperformed CellWhisperer (combined permutation test, *P =.003* and *P =.023*, respectively), confirming that both modalities contribute retrieval value beyond the text-only baseline.

**Table 2:** Combined permutation test across all 12 retrieval metrics and six datasets (50,000 sign-flip permutations; one-sided, H1: row > column).

**Method**	**versus Baseline**	** *p* (perm.)**	** *z* **	** *p* (analytic)**
Union (S+G)	CellWhisperer	$<2\times 10^{-5}$ ^***^	*4.95*	$4\times 10^{-7}$
Union (S+G)	BM25	$<2\times 10^{-5}$ ^***^	*5.11*	$2\times 10^{-7}$
Union (S+G)	Random	$<2\times 10^{-5}$ ^***^	*6.26*	$2\times 10^{-10}$
Semantic	CellWhisperer	*0.003* ^**^	*2.60*	$5\times 10^{-3}$
Semantic	BM25	*0.161*	*1.01*	*0.16*
Semantic	Random	$<2\times 10^{-5}$ ^***^	*5.22*	$9\times 10^{-8}$
scGPT	CellWhisperer	*0.023* ^*^	*1.96*	*0.025*
scGPT	BM25	*0.025* ^*^	*1.95*	*0.026*
scGPT	Random	$<2\times 10^{-5}$ ^***^	*6.09*	$6\times 10^{-10}$

ELISA Union significantly outperforms every baseline. All three Union comparisons exceed the permutation resolution and are reported as *p* < 2 × 10^−5^, the smallest value attainable with 50,000 permutations; the standardised effect size (*z*) and its analytic normal-approximation *p*-value resolve the ordering the censored permutation bound cannot, increasing from CellWhisperer (*z* = 4.95) through BM25 (*z* = 5.11) to Random (*z* = 6.26). The analytic approximation agrees with the exact permutation test on every comparison the latter can resolve (e.g. Semantic vs. BM25, *p* = 0.16 for both). Among single modalities, semantic retrieval does not significantly beat BM25, confirming BM25 is a strong lexical baseline on ontology queries; only the fused Union dominates on both axes. ^*^P <.05, ^**^P < .01, ^***^P < .001; *p* (analytic) from the normal approximation to the sign-flip null, reported as a complement to the exact permutation test.

A key observation is that no single retrieval modality dominated across both query types, which directly motivates the fusion design. The Semantic pipeline consistently excelled on ontology queries (mean MRR 0.879), where biological concept matching benefits from BioBERT’s language understanding, synonym expansion, and Cell Ontology name boosting, but fell to 0.491 on expression queries. The classical BM25 baseline showed the same profile strong on ontology (0.807) yet weak on expression (0.531) and was statistically indistinguishable from the Semantic pipeline overall (permutation *p = 0.16*), underscoring that BM25 is a genuinely competitive lexical baseline on concept queries rather than a straw man. In contrast, the gene marker scoring pipeline showed the inverse pattern, leading on expression queries (0.788) while underperforming on ontology axes (0.645), where matching transcriptomic signatures to cluster DE profiles is essential. This complementarity was particularly pronounced in the CF Airways dataset, where the Semantic pipeline achieved high ontology Recall@10 (*∼ *0.95) but lower expression recall, while the gene pipeline showed the inverse pattern. Similar modality-specific advantages were visible across all datasets: in the breast tissue atlas, semantic and union nearly overlapped on ontology metrics while the gene pipeline lagged; in ICB multi-cancer, the gene pipeline outperformed semantic on expression MRR while underperforming on ontology axes.

CellWhisperer showed competitive performance on ontology queries in several datasets, particularly CF Airways and High-Risk Neuroblastoma, where its ontology MRR approached that of the ELISA Semantic pipeline. However, CellWhisperer’s performance dropped substantially on expression queries across all six datasets, with a mean MRR of 0.397 *± * 0.049 compared with 0.806 *± * 0.061 for ELISA Union, a two-fold difference (Supplementary Table S1) [37, 38]. This gap was most severe in the ICB multi-cancer and first-trimester brain datasets, where CellWhisperer’s expression recall fell well below both ELISA pipelines. The expression query deficit reflects a fundamental architectural difference: CellWhisperer’s contrastive text-transcriptome alignment is optimized for natural-language cell type descriptions but does not incorporate a dedicated gene marker scoring mechanism for queries formulated as gene signatures, a query type that is common in exploratory single-cell analysis.

The ELISA Union mode resolves the tension between ontology and expression retrieval through its adaptive routing mechanism. For each query, the automatic classifier identifies whether the input is a gene list, a natural-language concept, or a mixture, and routes it to the appropriate pipeline. The additive union strategy then combines the full ranked output of the primary pipeline with unique clusters from the secondary pipeline, ensuring that relevant cell types captured by either modality are not lost. As a result, Union was the only mode ranked best on *both* axes (ontology MRR 0.922, expression MRR 0.806): in the CF Airways dataset, Union achieved a larger and more balanced radar footprint than any single modality; in the Breast Tissue Atlas, Union matched the near-perfect ontology performance of Semantic while substantially improving expression recall; and in the first-trimester brain, Union compensated for Semantic’s lower expression scores by incorporating the gene pipeline’s matching strength.

Notably, the performance advantage of ELISA was robust across datasets with very different structural properties. The CF Airways dataset (30 cell types, case-control design) and the first-trimester brain atlas (160 clusters, developmental trajectory without disease contrast) represent opposite ends of the complexity spectrum, yet ELISA Union outperformed every baseline in both settings. Similarly, the ICB Multi-Cancer dataset, which integrates nine cancer types across 223 patients, poses a challenging retrieval scenario owing to its heterogeneous cell type nomenclature, yet ELISA maintained its performance advantage.

In summary, ELISA’s hybrid retrieval architecture combining semantic language matching, gene marker scoring, and adaptive fusion provides a significantly superior retrieval framework compared with text-only multimodal approaches, classical lexical retrieval, and a random baseline (combined permutation test, $p < 2\times 10^{-5}$). The systematic advantage on expression queries, where dedicated gene scoring compensates for the limitations of language-only embeddings (Cohen’s *d* = 5.98 for MRR), together with the crossover whereby every single modality wins only one query type, establishes that both retrieval modalities contribute essential and non-redundant information for comprehensive single-cell atlas interrogation.

### ELISA replicates key biological findings across six diverse datasets

To evaluate whether ELISA could recover published biological conclusions through automated analysis alone, we compared ELISA-generated reports with the main-text results of six reference publications (Supplementary Table S5). For each dataset, ELISA was provided only with the preprocessed embedding file and no prior knowledge of the expected findings. We assessed replication across five quantitative metrics: gene coverage, pathway alignment, interaction recovery, proportion consistency, and theme coverage, and obtained an independent domain expert evaluation score (Table 3).

**Table 3:** Quantitative comparison between ELISA reports and reference single-cell studies.

**Dataset**	**Gene Cov.**	**Path. Align.**	**Int. Rec.**	**Prop.**	**Theme**	**Comp.**
	**(95% CI)**	**(95% CI)**	**(95% CI)**	**Cons.**	**Cov.**	**Score**
CF airway	0.80	1.00	0.20	Yes	0.85	0.71
	[0.66–0.89]	[0.70–1.00]	[0.04–0.62]			
Neuroblastoma	0.84	1.00	1.00	Yes	0.88	0.93
	[0.78–0.88]	[0.81–1.00]	[0.72–1.00]			
ICB multi-cancer	0.77	1.00	1.00	Yes	0.91	0.92
	[0.71–0.83]	[0.81–1.00]	[0.72–1.00]			
Breast atlas	0.96	1.00	0.80	Yes	0.89	0.91
	[0.92–0.98]	[0.84–1.00]	[0.49–0.94]			
Fetal lung AT2	1.00	1.00	0.40	Yes	0.88	0.82
	[0.96–1.00]	[0.76–1.00]	[0.12–0.77]			
Brain atlas	0.85	1.00	1.00	Yes	0.90	0.94
	[0.78–0.90]	[0.72–1.00]	[0.78–1.00]			
Mean	0.85	1.00	0.73	6/6	0.88	0.88
	[0.78–0.97]	[0.96–1.00]	[0.37–1.00]			
Pooled	844/960 recovered 0.879 95% CI [0.857–0.898]					

Gene Cov., gene coverage; Path. Align., pathway alignment; Int. Rec., interaction recovery; Prop. Cons., proportion consistency; Theme Cov., theme coverage. Comp. score, unweighted mean of the four continuous metrics (Prop. Cons. coded as 1.0 when consistent). Confidence intervals are 95% Wilson score intervals on the per-dataset recovery proportions *k/n*. Theme coverage is reported as the domain-expert score and is not a *k/n* proportion, so no interval is given. Mean-row intervals for gene coverage and interaction recovery are mean $\pm \,t\,$SE across the six datasets; the pathway-alignment mean interval is the pooled estimate (83/83), as all datasets attained *1.00*. The Pooled row aggregates all gene, pathway, and interaction benchmark items across the six datasets into a single recovery proportion.

Scores reflect agreement between ELISA-generated biological interpretations and findings described in the main text of the corresponding publications. Gene coverage, pathway alignment, interaction recovery, and proportion consistency were computed programmatically; theme coverage was assessed independently by a domain expert as described in Supplementary Section S9. For the three programmatically computed recovery proportions, bracketed ranges give 95% Wilson score confidence intervals reflecting the number of reference items (*n*) evaluated per dataset. Wide intervals on the interaction metric reflect the small number of benchmark interactions available per individual study; the pooled estimate across all datasets (final row) is correspondingly tighter. Raw recovery counts are given in Supplementary Table S4.

Across all six datasets, ELISA achieved a mean composite score of 0.88 (range 0.82–0.96). Pathway alignment and theme coverage were near-perfect (mean 1.0 each), while gene coverage averaged 0.85 and interaction recovery 0.73. Independent biological evaluation scores (mean 0.88) confirmed strong agreement with published findings. The computation of these metrics is presented in Supplementary Section S7.

#### Airways with cystic fibrosis

ELISA was used to recover the major epithelial and immune cell populations, as described by Berg *et al.* [22], including correct proportion shifts and IFN-*γ */type I interferon programs (pathway alignment: 1.0). Gene coverage reached 0.80, capturing markers such as *IFNG*, *CD69*, and *HLA-E*. Interaction recovery was 0.20, reflecting partial detection of the HLA-E/NKG2A and CALR–LRP1 axes (composite: 0.82).

#### High-risk neuroblastoma

ELISA identified all major cellular compartments and correctly detected the HB-EGF/ERBB4 paracrine axis (interaction recovery: 1.00) as described by Yu *et al.* [23]. Pathway alignment was perfect and with mTOR, MAPK, and ErbB programs identified. Gene coverage was 0.84, with partial recovery of therapy-induced markers (composite: 0.95).

#### Immune checkpoint blockade across cancers

Using the ICB dataset, Gondal *et al.* [24], ELISA captured checkpoint molecules (*CD274*, *PDCD1*, *andCTLA4*), exhaustion markers, and all major LR axes including PD-L1/PD-1 and TIGIT/NECTIN2 (gene coverage: 0.73; pathway and interaction recovery: 1.00; composite: 0.93).

#### Healthy breast tissue atlas

ELISA achieved its highest composite score (0.96) on the dataset of Bhat-Nakshatri *et al.* [26], accurately resolving the epithelial hierarchy with a gene coverage of 0.96, perfect pathway alignment, and interaction recovery of 0.80. Ancestry-related transcriptional programs were not captured, reflecting a limitation of ELISA’s pathway-centric framework.

#### Fetal lung Alveolar Type (AT2) organoids

ELISA achieved perfect gene coverage (1.00) on the dataset of Lim *et al.* [25], detecting all canonical surfactant genes and correctly identifying surfactant metabolism, Wnt, and Fibroblast Growth Factor (FGF) programs. Interaction recovery was lower (0.40), as SFTPC trafficking mechanisms were outside transcriptomic scope (composite: 0.91).

#### First-trimester human brain

Despite operating solely on the transcriptomic component of this multimodal atlas [27], ELISA identified major neuronal populations with gene coverage of 0.85 and perfect pathway and interaction recovery. Chromatin accessibility analyses were correctly identified as outside scope (composite: 0.95).

#### Summary

ELISA demonstrated robust replication across all six datasets (mean composite 0.88), with the strongest performance for pathway-level and thematic interpretation (*≥ *0.88 mean). Gene coverage was high but not exhaustive (0.85), with missed genes primarily in rare cell states and non-transcriptomic modalities. To quantify the uncertainty of these recovery metrics, we computed 95% Wilson score confidence intervals for each programmatically evaluated proportion (gene coverage, pathway alignment, and interaction recovery; Table 3, Supplementary Section S5). The width of each interval is governed by the number of reference items available per dataset: gene coverage, evaluated over the largest reference sets (45–206 genes), is estimated most precisely, whereas interaction recovery is evaluated over far fewer items (5–14 interactions per study) and therefore carries correspondingly wider intervals. The wide intervals on this metric thus reflect the small number of LR interactions reported in the individual reference studies rather than instability in ELISA’s predictions, which are deterministic given a fixed embedding. Aggregating across all six datasets, ELISA recovered *844/960* benchmark items, an overall recovery of *0.879* (95% CI *[0.857,0.898]*), and pooled interaction recovery was *45/54=0.833* (95% CI *[0.713,0.910]*).

### Discovery of candidate regulatory signals across tissue atlases

Beyond reproducing the key biological signals described in the original studies, ELISA’s discovery mode highlighted several candidate regulatory signals that were not explicitly emphasized in the reference publications (Table 4). These signals represent transcriptome-derived hypotheses emerging from systematic cross-cell-type analysis of single-cell atlases.

**Table 4:** Candidate regulatory signals identified by ELISA's discovery mode across six reference single-cell atlases, representing transcriptome-derived hypotheses not explicitly highlighted in the original publications.

**Dataset**	**Primary finding in reference study**	**ELISA candidate discovery / hypothesis**
CF airway	Altered immune–structural cell crosstalk and inflammatory signaling in CF airway tissue	Detection of the macrophage *CALR–LRP1* signaling axis, suggesting altered apoptotic cell recognition or phagocytic clearance pathways contributing to the CF lung inflammatory microenvironment
Breast atlas	Ancestry-associated epithelial lineage variation and luminal progenitor states in healthy breast tissue	Enrichment of the Kelch-family gene *KLHL29* in basal–myoepithelial cells, suggesting a potential additional marker or regulator of basal epithelial structural identity
Fetal lung AT2	ITCH-mediated ubiquitin-dependent regulation of surfactant protein C (SFTPC) maturation in alveolar type II cells	Upregulation of *TRIM21* and *TRIM65* in mature AT2 cells, suggesting additional TRIM-family ubiquitin ligases may participate in surfactant protein processing and proteostasis
ICB multi-cancer	Tumor and immune transcriptional responses associated with ICB therapy	Elevated *CD163* and *MRC1* expression in tumor-associated macrophages, consistent with an M2-like polarization state potentially associated with therapy-induced immune remodeling
Neuroblastoma	Therapy-induced transcriptional rewiring of tumor cell states and microenvironment interactions	Differential AP-1 transcription factor usage, with *JUND* enriched at diagnosis and *JUNB/FOS* enriched post-treatment, suggesting stress-response remodeling during therapy-induced state transitions
Brain development atlas	Chromatin accessibility programs defining early neuronal lineage specification	Shared transcription factor module (*TFAP2B*, *LHX5*, and*LHX1*) across Purkinje neurons and midbrain GABAergic populations, suggesting a conserved regulatory program for inhibitory neuron specification

In the CF airway dataset, ELISA identified enrichment of the *CALR–LRP1* phagocytic signaling axis within the macrophage populations. Calreticulin–LRP1 signaling has previously been implicated in apoptotic cell recognition and clearance, suggesting that altered macrophage-mediated phagocytosis may contribute to the inflammatory microenvironment characteristic of the CF lung.

Within the fetal lung atlas, ELISA detected increased expression of the ubiquitin-associated regulators *TRIM21* and *TRIM65* in alveolar type II (AT2) cells alongside the known E3 ubiquitin ligase *ITCH*. Although *ITCH* has been implicated in regulating surfactant protein C (SFTPC) maturation, the enrichment of these additional TRIM-family ligases suggests that cooperative ubiquitin-dependent pathways may participate in surfactant protein processing and AT2 cell proteostasis.

In the healthy breast tissue atlas, ELISA highlighted strong enrichment of the Kelch-family gene *KLHL29* within basal–myoepithelial cell populations. Although not emphasized in the original study, this pattern suggests that *KLHL29* may represent a previously unrecognized marker or structural regulator of basal epithelial identity.

Analysis of the ICB dataset revealed elevated expression of macrophage markers *CD163* and *MRC1* within tumor-associated macrophage populations following therapy. This expression pattern is consistent with an M2-like macrophage polarization state, potentially reflecting remodeling of the immune microenvironment in response to checkpoint blockade treatment.

In the neuroblastoma dataset, ELISA identified differential usage of AP-1 transcription factors across treatment states. Specifically, *JUND* expression was enriched at diagnosis, whereas *JUNB* and *FOS* were more strongly expressed after therapy. This shift suggests dynamic remodeling of AP-1–mediated stress-response programs during therapy-induced tumor state transitions.

Finally, analysis of the developing brain atlas revealed a shared transcription factor module composed of *TFAP2B*, *LHX5*, and *LHX1* across Purkinje neurons and midbrain GABAergic neuronal populations. This co-occurring regulatory signature suggests the existence of a conserved transcriptional program underlying inhibitory neuron specification in anatomically distinct brain regions.

Taken together, these findings illustrate how ELISA can surface candidate regulatory programs across diverse single-cell atlases. These signals should be interpreted as transcriptome-derived hypotheses and may serve as starting points for targeted experimental validation.

## Discussion

In this study, we introduced ELISA, an agent-based framework that unifies semantic language retrieval, gene marker scoring, and LLM-mediated biological interpretation for interactive single-cell atlas interrogation. Systematic evaluation across six diverse datasets demonstrated that ELISA significantly outperforms CellWhisperer in cell type retrieval (combined permutation test, $p<2\times 10^{-5}$) and faithfully replicated published biological findings with a mean composite score of 0.88. Here, we discuss the implications of these results for the design of retrieval systems in single-cell genomics, the limitations of contrastive multimodal alignment, and the broader role of agentic AI in biological discovery.

### Contrastive alignment produces text-dominated embeddings

A central finding of this study is the striking asymmetry in CellWhisperer performance across query types. In ontology queries natural language descriptions of cell types and biological processes CellWhisperer performed competitively with ELISA’s Semantic pipeline, achieving mean ontology MRR values within 0.15 of ELISA Union across most datasets (Supplementary Table S1, Fig. 2). This is expected: CellWhisperer’s CLIP-style contrastive training aligns transcriptome embeddings with textual descriptions, and ontology queries directly exploit this text-side alignment. However, on expression queries where users provide gene signatures rather than natural language CellWhisperer’s performance collapsed, with expression MRR averaging 0.397 compared with 0.806 for ELISA Union, a two-fold deficit (Cohen’s *d* = 5.98).

This asymmetry reveals a fundamental limitation of contrastive multimodal alignment for single-cell retrieval. CLIP-style training optimizes for text transcriptome correspondence by learning a shared embedding space where matching text cell pairs are close and mismatched pairs are distant. The resulting embeddings are, by construction, shaped primarily by the textual supervision signal: the model learns to position transcriptomes near their text descriptions, but the fine-grained transcriptomic structure which genes are differentially expressed, at what fold changes, in what fraction of cells is compressed into a representation optimized for text matching rather than gene-level querying. When a user submits a gene signature such as “MARCO FABP4 APOC1 C1QB C1QC MSR1”, these gene names are processed as text tokens rather than matched against DE statistics, resulting in a retrieval signal that is weaker and less specific than direct marker scoring.

This observation has broader implications than those of ELISA and CellWhisperer. As foundation models for single-cell biology increasingly adopt contrastive or multimodal pretraining objectives, our results caution that text-supervised alignment may inadvertently sacrifice expression-level specificity. The dual-query evaluation framework introduced here requiring systems to perform well on both ontology and expression queries provides a principled diagnostic for detecting such modality imbalances.

### Explicit routing outperformed implicit fusion

ELISA’s architectural response to this challenge was to avoid implicit embedding fusion altogether. Rather than learning a single shared space that must simultaneously serve text and expression queries, ELISA maintains two separate representation spaces BioBERT semantic embeddings and gene-level DE statistics, and routes queries to the appropriate pipeline through explicit classification. The query classifier, operating on simple token-level heuristics (gene name patterns, known vocabulary membership, and natural language indicators), achieved reliable routing across all six datasets without requiring any training data.

This design choice is supported empirically by complementarity analysis: the semantic pipeline won ontology queries, while the gene marker scoring pipeline won on expression queries in every dataset, with minimal overlap in their error profiles. The additive union strategy, which selects the better-performing modality as the primary and appends unique results from the secondary, captures the strengths of both pipelines without the compression artifacts inherent in learned fusion. The result was a system that matched or exceeded the best single modality on every metric across every dataset a property that no implicit fusion method could guarantee.

### Analytical modules bridge retrieval and interpretation

A distinguishing feature of ELISA relative to prior retrieval-focused systems is the integration of downstream analytical modules pathway scoring, LR interaction prediction, comparative analysis, and proportion estimation that operate directly on the same embedded data representation used for retrieval. This design enables a seamless transition from “which cell types are relevant?” (retrieval) to “what biological programs are active in these cell types?” (analysis) to “what does this mean biologically?” (LLM interpretation), all within a single interactive session.

The perfect pathway alignment (mean 1.00) and high theme coverage (mean 0.88) scores across all six datasets demonstrated that this integrated architecture effectively connects gene-level evidence to biological programs. In contrast, systems that perform retrieval alone including CellWhisperer, require users to manually extract gene lists from retrieved clusters and perform separate pathway and interaction analyses using external tools, introducing friction and potential inconsistencies.

The interaction recovery metric (mean 0.73) was the most variable across datasets, with perfect recovery in neuroblastoma, ICB, and brain datasets but lower recovery in CF (0.20) and fetal lung (0.40). These lower scores primarily reflect the inherent difficulty of predicting specific LR pairs from expression data when the ligand or receptor is expressed at moderate levels across multiple cell types, making the interaction statistically detectable but not highly ranked. Future work could address this by incorporating spatial proximity information or protein-level data to improve the interaction specificity.

### Large-language model grounding and the discovery hallucination boundary

ELISA’s discovery mode, which prompts the LLM to separate dataset evidence from established biology and to propose hypotheses with probabilistic language, generated biologically plausible candidate signals in all six datasets (Table 4). These include the CALR/LRP1 phagocytic axis in CF macrophages, differential AP-1 family member usage in neuroblastoma therapy response, and a shared TFAP2B/LHX5/LHX1 regulatory module across inhibitory neuron subtypes in the developing brain. While these hypotheses require experimental validation, they illustrate the potential of grounded LLM reasoning to surface non-obvious patterns in complex datasets.

However, a strict separation between data-derived evidence and LLM-generated interpretation is essential. Without it, the LLM would inevitably introduce plausible-sounding but unsupported claims a risk that is particularly acute in biology, where prior knowledge is vast and contextual. ELISA’s prompt architecture addresses this by providing the LLM only with retrieved cluster data, gene statistics, and pathway results as context, with explicit instructions to avoid external literature and causal claims.

### Future directions

Several extensions can strengthen and broaden ELISA’s capabilities. Integration with spatial transcriptomics data would enable spatially resolved interaction prediction, addressing the current limitation of expression-only interaction scoring. Incorporation of trajectory inference methods would allow ELISA to reason about dynamic processes such as differentiation and therapy response. Expansion of the retrieval engine to support cross-dataset queries comparing cell types across tissues or disease states would enable the kind of meta-analytical reasoning that was outside ELISA’s scope in the ICB dataset evaluation. Finally, replacing the fixed LLM with a fine-tuned model trained on single-cell biological reasoning can improve the specificity and depth of automated interpretations. Beyond these, extending ELISA past human, droplet-based data to other species via species-appropriate foundation models and to additional platforms such as plate-based and spatial assays is a key direction for broadening its applicability; we discuss the architectural basis for this generalization in Supplementary Section S12.

### Computational efficiency and scalability

ELISA decouples a one-time, offline embedding step from online querying. Embedding (scGPT and BioBERT) is computed once per dataset; queries then operate on the cluster-level .pt representation, so query latency scales with the number of clusters (tens), not cells. On our largest dataset (${\sim }372{\,}000$ cells), embedding required ${\sim }4$ h on a single A100 (80 GB), after which a query returns in *1*–*2* s and a *100*-query report in ${\sim }30$ min (no GPU needed). The embedding step is linear in cell number (${\sim }30$ cells s$^{-1}$), so a one-million-cell atlas is projected at ${\sim }9$ h of overnight preprocessing, while query cost stays constant. The initial embedding of very large atlases remains the dominant, GPU-bound cost and is the main limitation; further detail is given in Supplementary Section S2.

## Conclusion

ELISA demonstrates that explicit modality routing, rather than implicit contrastive fusion, provides a more robust foundation for multimodal single-cell retrieval. By maintaining separate semantic and expression pipelines and combining them through adaptive query classification, ELISA achieves consistently superior performance across both natural language and gene-signature queries. The integration of analytical modules and grounded LLM interpretation within a single interactive framework bridges the gap between data exploration and biological discovery, enabling researchers to move from raw atlas data to structured biological hypotheses within a single session. As single-cell datasets continue to grow in scale and complexity, systems that combine the complementary strengths of language models and expression-aware retrieval will be essential for translating transcriptomic data into biological understanding.

**Key Points**

- ELISA (Embedding-Linked Interactive Single-cell Agent) is an interpretable AI agent that unifies single-cell generative pretrained transformer expression embeddings with biomedical bidirectional encoder representations from transformers-based semantic retrieval and large language model-mediated reasoning for natural-language-driven single-cell genomics discovery.
- An automatic query classifier dynamically routes gene-signature, natural-language, and mixed queries to dedicated retrieval pipelines, enabling flexible navigation of complex cellular landscapes.
- ELISA significantly outperforms CellWhisperer in cell-type retrieval across six diverse single-cell RNA sequencing datasets (combined permutation test, $p<2\times 10^{-5}$), with particularly large gains on gene-signature queries (Cohen’s *d = 5.98* for mean reciprocal rank).
- Integrated analytical modules for pathway scoring, ligand–receptor interaction prediction, comparative analysis, and proportion estimation operate directly on embedded data without requiring access to the original count matrix.
- ELISA replicates published biological findings with high fidelity (mean composite score 0.88) and generates grounded candidate hypotheses, bridging the gap between transcriptomic data exploration and biological discovery.

## Supplementary Material

ElisaOmarSupplementary_bbag501

elisa_report_CysticFibrosis_bbag501

## Data Availability

All six scRNA-seq datasets used in this study are publicly available through CZ CELLxGENE Discover (https://cellxgene.cziscience.com): CF airways [22], high-risk neuroblastoma [23], ICB multi-cancer [24], fetal lung AT2 organoids [25], healthy breast tissue [26], and first-trimester brain [27]. Datasets were downloaded in AnnData (.h5ad) format. Source code is available at https://github.com/omaruno/ELISA-An-AI-Agent-for-Expression-Grounded-Discovery-in-Single-Cell-Genomics .

## Abbreviations

scRNA-seq, single-cell RNA sequencing; ELISA, Embedding-Linked Interactive Single-cell Agent; scGPT, single-cell Generative Pre-trained Transformer; BioBERT, Biomedical Bidirectional Encoder Representations from Transformers; LLM, large language model; MRR, Mean Reciprocal Rank; RRF, reciprocal rank fusion; DE, differential expression; LR, ligand–receptor; GO, Gene Ontology; RAG, retrieval-augmented generation
