# Supplementary material for: ELISA (Embedding-Linked Interactive Single-cell Agent): an interpretable hybrid generative Artificial Intelligence agent for expression-grounded discovery in single-cell genomics
Source: Brief Bioinform. 2026 Sep 10;27(5):bbag501. doi: 10.1093/bib/bbag501 (PMC13561307; doi:10.1093/bib/bbag501)
Supplement: Supplementary file 1 [file elisaomarsupplementary_bbag501.pdf]

Supplementary Material

ELISA: An Interpretable Hybrid Generative AI Agent for Expression-Grounded Discovery in Single-Cell Genomics

Omar Coser

25/08/2026

Contents

S1 ELISA produces auditable, provenance-grounded interpretations 3

S2 Computational efficiency and scalability 3

S3 Software and reproducibility 4

S4 Full statistical comparison across all baselines 4

S5 Statistical confidence for replication metrics 4

S6 Discovery-mode report generation 7

S7 Replication evaluation metrics 8

S8 Retrieval and analytical evaluation metrics 9
- S8.1 Retrieval metrics . . . 9
- S8.2 Statistical testing . . . 10

S9 Human evaluation protocol 10

S10 Materials: datasets 11

S11 Methods 12
- S11.1 ELISA: architecture and design principles . . . 12
- S11.2 Hybrid retrieval engine . . . 13
  - S11.2.1 Query classification and routing . . . 13
  - S11.2.2 Gene marker scoring pipeline . . . 13
  - S11.2.3 Semantic matching pipeline . . . 14
  - S11.2.4 Reciprocal rank fusion for mixed queries . . . 14
  - S11.2.5 Additive union evaluation strategy . . . 14
- S11.3 Analytical modules . . . 14
  - S11.3.1 Cell–cell interaction prediction . . . 14
  - S11.3.2 Pathway activity scoring . . . 15
  - S11.3.3 Comparative analysis . . . 15
  - S11.3.4 Proportion analysis . . . 15
  - S11.3.5 Additional analytical functions . . . 15
- S11.4 Visualization module . . . 15
- S11.5 LLM-mediated chat interface . . . 16
- S11.6 Benchmarking framework . . . 16

S11.6.1 Query design . . . 16
S11.6.2 Baseline comparisons . . . 16
S11.6.3 Metrics . . . 16
S11.6.4 Analytical module evaluation . . . 17
S11.7 Data representation and preprocessing . . . 17
S11.8 Software dependencies and reproducibility . . . 17

S12 Extended future directions: generalizability across technologies, modalities, and species **17**

S13 ELISA parameters and hyperparameters **18**

S14 Benchmark queries **22**
S14.1 D1: Cystic Fibrosis Airways (Berg *et al.* [1]) . . . 22
S14.1.1 Ontology queries . . . 22
S14.1.2 Expression queries . . . 23
S14.2 D2: High-Risk Neuroblastoma (Yu *et al.* [2]) . . . 25
S14.2.1 Ontology queries . . . 25
S14.2.2 Expression queries . . . 27
S14.3 D3: Immune Checkpoint Blockade Multi-Cancer (Gondal *et al.* [3]) . . . 28
S14.3.1 Ontology queries . . . 28
S14.3.2 Expression queries . . . 30
S14.4 D4: Fetal Lung AT2 Organoids (Lim *et al.* [4]) . . . 32
S14.4.1 Ontology queries . . . 32
S14.4.2 Expression queries . . . 33
S14.5 D5: Healthy Breast Tissue Atlas (Bhat-Nakshatri *et al.* [5]) . . . 35
S14.5.1 Ontology queries . . . 35
S14.5.2 Expression queries . . . 37
S14.6 D6: First-Trimester Human Brain (Mannens *et al.* [6]) . . . 38
S14.6.1 Ontology queries . . . 38
S14.6.2 Expression queries . . . 40

S15 Example visualizations: cystic fibrosis dataset **42**

S1 ELISA produces auditable, provenance-grounded interpretations

A central design goal of ELISA is that every generated statement can be traced back to the transcriptomic evidence that produced it. ELISA does not expose the internal weights or attention of the language model, which is accessed as a fixed reasoning layer; rather, it is interpretable by construction, because each stage of the pipeline reports an explicit numerical score and the language model receives only this dataset-derived evidence as context. We illustrate this with the HLA-E/NKG2A immune-checkpoint axis in cystic fibrosis, traced end to end through the four stages of the system.

At the *retrieval* stage, the query "HLA-E NKG2A immune checkpoint and CD8 T cell inhibition in CF" is matched against the BioBERT cluster-description embeddings; the top-ranked clusters and their relevance scores (cosine similarity between the L2-normalised query and cluster embeddings, augmented by a Cell Ontology name-boost term) are cytotoxic T cell (0.684), $CD4^+$ helper T cell (0.625) and natural killer cell (0.602) the cytotoxic and NK populations that bear the NKG2A receptor. At the *interaction* stage, the module scores the HLA-E/KLRD1 (NKG2A) pair at 0.688 and recovers HLA-E→KLRC1 and HLA-E→KLRC2 as full matches against the reference ground truth. At the *evidence* stage, the comparative module exposes the differential-expression values underlying the axis (CF vs. control): the NKG2A receptor genes are strongly upregulated in precisely the retrieved cells (KLRD1: NK +5.07, CD8 +3.87; KLRC1: CD8 +3.98, cytotoxic T +4.66 in $\log_2$ fold-change), with concomitant upregulation of the HLA-E ligand (NK +2.00, CD8 +1.49). Finally, at the *interpretation* stage, the language model receiving only this evidence summarises the axis as being characterised by the upregulation of HLA-E and KLRC1, naming exactly the genes upregulated in the data.

Every layer therefore agrees, and each is quantified: the retrieval scores, the interaction score, and the differential-expression values can all be inspected independently. Interpretability in ELISA is thus a property of provenance rather than of model-internal introspection any claim in the final report can be followed back to an explicit retrieval score and a differential-expression value. A further consequence of this design is that the language model, being restricted to the retrieved evidence, hedges when that evidence is weak rather than fabricating support, abstaining instead of confabulating when a proposed mechanism is not represented in the data.

S2 Computational efficiency and scalability

ELISA decouples a one-time, offline embedding step from online querying, which determines its computational profile. The embedding step scGPT expression embeddings and BioBERT semantic embeddings is performed once per dataset, after which all queries operate on the resulting cluster-level representation (the serialised `.pt` file) rather than on individual cells. Query latency therefore scales with the number of clusters (typically tens) and is effectively independent of the number of cells in the atlas.

On our largest dataset (high-risk neuroblastoma, ∼372,000 cells), the one-time embedding step required ∼3.45 h for scGPT and ∼0.5 h for the BioBERT semantic stage on a single NVIDIA A100 (80 GB), i.e. approximately four hours of offline preprocessing. Because cells are processed in fixed mini-batches, peak GPU memory is bounded by the batch size and does not grow with dataset size; smaller GPUs can be accommodated by reducing the batch size. After preprocessing, retrieval and analysis require no GPU: a single query returns in under 1–2 s, and a full 100-query report including LLM-mediated interpretation completed in ∼30 min, with per-query latency dominated by the language-model API call rather than by ELISA's retrieval or analysis.

The embedding step is linear in the number of cells (∼30 cells s^{-1} for scGPT on the A100), so a one-million-cell atlas is projected to require ∼9 h of one-time embedding within an overnight run while query-time latency and memory remain essentially constant as the atlas grows. This embedding cost is inherited from the underlying foundation model (scGPT, pretrained on over

33 million cells [7]) and is shared by any scGPT-based pipeline; being an embarrassingly parallel, batched operation, it can be distributed across GPUs for further speed-up. A current limitation is therefore that, although querying is lightweight and scales to large atlases, the initial embedding of very large datasets remains GPU-bound and constitutes the dominant computational cost; reducing this cost (e.g. through embedding caching or lighter-weight expression encoders) is a direction for future work.

S3 Software and reproducibility

ELISA was implemented in Python 3.10+ using PyTorch, sentence-transformers [8], scanpy [9], scikit-learn, and UMAP-learn [10]. All retrieval and analytical steps were performed on a standard workstation without GPU requirements; the one-time embedding step was performed on an NVIDIA A100 GPU with 80 GB of memory. Source code, benchmark queries, and evaluation scripts are available at `https://github.com/omaruno/ELISA-An-AI-Agent-for-Expression-Grounded-Disc` Use of an LLM (LLaMA-3.1-8B) for automated interpretation is documented in accordance with journal policy.

S4 Full statistical comparison across all baselines

This section reports the complete per-metric statistical comparison of ELISA Union against all three baselines (CellWhisperer, BM25, and Random) across the six benchmark datasets. Table S1 gives the Union-vs-CellWhisperer comparison summarised in the main text; Tables S2 and S3 give the corresponding Union-vs-BM25 and Union-vs-Random results.

BM25 [11] was computed over the same cluster text representations used by the semantic pipeline (cell-type name, Cell Ontology terms, and top marker genes), using the standard parameterisation $k_1 = 1.5$, $b = 0.75$. It was evaluated with the identical query sets, expected-cluster ground truth, and metrics as all other retrieval modes, so the comparison is exactly paired across the six datasets. All p-values are one-sided paired t-tests (H_1: Union > baseline); W/L counts the datasets on which Union won/lost. Metrics with $n < 6$ reflect the different Recall@k cutoffs used per dataset. With $n = 6$ the paired Wilcoxon signed-rank floor is $p = 0.016$, which is why the combined permutation test (Table 2 of the main text) provides the most discriminating significance estimate.

Table S1 details the comparison against CellWhisperer, the primary state-of-the-art reference. Union's advantage is largest on expression queries (mean ΔMRR = +0.41, $d = 5.98$) and remains positive on every ontology metric, with no metric favouring CellWhisperer.

Table S2 compares Union against the classical lexical baseline (BM25). BM25 is competitive on ontology queries (cf. Table 2 of the main text), yet Union improves significantly on both axes, with the gains on expression MRR ($\Delta = +0.28$) and ontology MRR ($\Delta = +0.11$) significant at $n = 6$.

Table S3 reports the comparison against a random-ranking floor, included as a sanity check. As expected, Union exceeds random ranking by a wide margin on every metric, with the largest effect sizes of the three comparisons.

S5 Statistical confidence for replication metrics

We provide statistical confidence for the quantitative replication metrics reported in Table 3 of the main text. The pathway alignment, interaction recovery, and gene coverage metrics are each recovery proportions: a count k of reference items recovered by ELISA out of a total n of items reported in the corresponding reference publication. Because each metric is a proportion estimated from a finite number of reference items, we quantify its uncertainty with a 95% Wilson score

Table S1: Statistical comparison of ELISA Union vs. CellWhisperer retrieval performance. For each metric, Δ mean reports the average improvement of Union over CellWhisperer across datasets. Cohen's d is the paired effect size. p-values are from one-sided paired t-tests (H_1: Union > CellWhisperer). W/L counts datasets where Union won/lost. Metrics with fewer than 6 datasets reflect different Recall@k cutoffs used per dataset (see Section S8). The combined permutation test aggregates all metrics simultaneously.

Category	Metric	Δ mean	Cohen's d	p (paired t)	W/L	n
Expression	MRR	+0.409	5.98	<0.001	6/0	6
Expression	Recall@5	+0.287	1.57	0.006	5/0	5
Expression	Recall@3	+0.428	5.38	0.006	3/0	3
Expression	Recall@2	+0.492	3.43	0.014	3/0	3
Expression	Recall@1	+0.442	1.84	0.043	3/0	3
Expression	Recall@10	+0.284	1.43	0.065	3/0	3
Ontology	MRR	+0.152	1.02	0.028	5/1	6
Ontology	Recall@5	+0.078	0.84	0.047	4/1	5
Ontology	Recall@10	+0.113	2.46	0.025	3/0	3
Ontology	Recall@1	+0.086	0.61	0.199	2/1	3
Ontology	Recall@2	+0.046	0.73	0.166	2/1	3
Ontology	Recall@3	+0.032	0.80	0.150	2/1	3
Combined (all 12 metrics)		permutation $p < 2 \times 10^{-5\dagger}$				6

†Combined permutation test, 50,000 permutations.

Table S2: ELISA Union vs. BM25 — per-metric statistical tests. Columns as in Table S1.

Category	Metric	Δ mean	Cohen's d	p (paired t)	W/L	n
Expression	MRR	+0.275	1.56	0.006	6/0	6
Expression	Recall@5	+0.233	0.91	0.038	5/0	5
Expression	Recall@3	+0.298	1.26	0.081	3/0	3
Expression	Recall@2	+0.412	2.46	0.026	3/0	3
Expression	Recall@1	+0.317	1.17	0.090	3/0	3
Expression	Recall@10	+0.158	1.11	0.097	3/0	3
Ontology	MRR	+0.114	2.03	0.002	6/0	6
Ontology	Recall@5	+0.103	1.20	0.016	5/0	5
Ontology	Recall@10	+0.069	1.69	0.050	3/0	3
Ontology	Recall@1	+0.187	2.63	0.022	3/0	3
Ontology	Recall@2	+0.212	4.67	0.008	3/0	3
Ontology	Recall@3	+0.163	3.43	0.014	3/0	3
Combined (all 12 metrics)		permutation $p < 2 \times 10^{-5}$				6

Table S3: ELISA Union vs. Random — per-metric statistical tests. Columns as in Table S1.

Category	Metric	Δ mean	Cohen's d	p (paired t)	W/L	n
Expression	MRR	+0.460	7.82	<0.001	6/0	6
Expression	Recall@5	+0.351	1.67	0.005	5/0	5
Expression	Recall@3	+0.418	7.38	0.003	3/0	3
Expression	Recall@2	+0.490	5.58	0.005	3/0	3
Expression	Recall@1	+0.453	4.08	0.010	3/0	3
Expression	Recall@10	+0.335	2.44	0.026	3/0	3
Ontology	MRR	+0.597	4.70	<0.001	6/0	6
Ontology	Recall@5	+0.440	1.64	0.005	5/0	5
Ontology	Recall@10	+0.451	12.55	0.001	3/0	3
Ontology	Recall@1	+0.641	5.92	0.005	3/0	3
Ontology	Recall@2	+0.579	4.07	0.010	3/0	3
Ontology	Recall@3	+0.480	2.61	0.023	3/0	3
Combined (all 12 metrics)		permutation $p < 2 \times 10^{-5}$				6

confidence interval, which is appropriate for binomial proportions and, unlike the normal (Wald) approximation, remains within $[0, 1]$ even for small n or proportions near the boundary.

For a metric with k successes in n trials and observed proportion $\hat{p} = k/n$, the Wilson score interval at confidence level $1 - \alpha$ is

$$\frac{\hat{p} + \dfrac{z^2}{2n} \pm z\sqrt{\dfrac{\hat{p}(1-\hat{p})}{n} + \dfrac{z^2}{4n^2}}}{1 + \dfrac{z^2}{n}}, \tag{S1}$$

where $z = 1.96$ for a 95% interval. We applied this to each programmatically computed recovery metric per dataset; the resulting intervals are reported alongside the point estimates in Table 3 of the main text, and the underlying raw counts k/n are given in Table S4.

Per-dataset intervals. Gene coverage, which is evaluated over the largest number of reference items (45–206 key genes per dataset), is estimated most precisely: intervals span at most ± 0.08 and are as tight as $[0.92, 0.98]$ for the breast atlas (170/177). Pathway alignment is 1.00 in every dataset; the corresponding intervals (e.g. $[0.70, 1.00]$ for 9/9, $[0.84, 1.00]$ for 20/20) reflect that a perfect score over a finite reference set is still consistent with a true rate somewhat below one. Interaction recovery is evaluated over the smallest reference sets (5–14 interactions per dataset) and therefore carries the widest intervals: for example, the cystic fibrosis dataset (1/5) yields 0.20 with a 95% interval of $[0.04, 0.62]$, and the fetal lung dataset (2/5) yields 0.40 with $[0.12, 0.77]$. These wide intervals are an honest consequence of the limited number of ligand–receptor interactions reported in the individual reference studies, rather than of instability in ELISA's predictions, which are deterministic given a fixed embedding.

Pooled estimates. Because the per-dataset interaction reference sets are small, we additionally report pooled estimates that aggregate the recovered and total items across all six datasets for each metric. Pooled gene coverage is $716/823 = 0.870$ ($[0.845, 0.891]$), pooled pathway alignment is $83/83 = 1.000$ ($[0.956, 1.000]$), and pooled interaction recovery is $45/54 = 0.833$ ($[0.713, 0.910]$). Considering all gene, pathway, and interaction benchmark items together, ELISA recovered 844/960 items, an overall recovery of 0.879 with a 95% Wilson confidence interval of $[0.857, 0.898]$. The pooled interaction estimate (0.833) is substantially more precise than any individual dataset and indicates that, while interaction recovery varies across studies owing to small per-study reference sets, the aggregate performance is high and tightly bounded.

Scope. These intervals are reported for the three programmatically computed recovery proportions (gene coverage, pathway alignment, interaction recovery). Proportion consistency is a binary per-dataset directional criterion (passed in all six datasets, 6/6) and is therefore summarised as a count of passing datasets rather than a per-item proportion. Theme coverage in Table 3 of the main text is the independent domain-expert score described in Section S9; as it is a holistic expert rating rather than a k/n count, no binomial interval is attached to it. The programmatic keyword-presence counts for theme coverage are nonetheless tabulated for completeness in Table S4.

Table S4: Raw recovery counts underlying Table 3 of the main text. Each entry gives the number of recovered reference items over the total evaluated (k/n) together with the corresponding 95% Wilson score confidence interval. These counts are the data from which the proportions and intervals in Table 3 of the main text are derived. Proportion consistency is a binary directional criterion and is summarised as the number of datasets passing. Theme coverage is shown here as its programmatic keyword-presence count; the value reported in Table 3 of the main text is instead the independent domain-expert score (see footnote).

Dataset	Gene Cov. k/n (95% CI)	Path. Align. k/n (95% CI)	Int. Rec. k/n (95% CI)	Prop. Cons.	Theme Cov. k/n (95% CI)	Comp. score
CF airway	36/45 [0.66–0.89]	9/9 [0.70–1.00]	1/5 [0.04–0.62]	Yes	8/8 [0.68–1.00]	0.71
Neuroblastoma	173/206 [0.78–0.88]	16/16 [0.81–1.00]	10/10 [0.72–1.00]	Yes	24/24 [0.86–1.00]	0.93
ICB Multi-Cancer	133/172 [0.71–0.83]	16/16 [0.81–1.00]	10/10 [0.72–1.00]	Yes	20/20 [0.84–1.00]	0.92
Breast Atlas	170/177 [0.92–0.98]	20/20 [0.84–1.00]	8/10 [0.49–0.94]	Yes	29/29 [0.88–1.00]	0.91
Fetal Lung AT2	97/97 [0.96–1.00]	12/12 [0.76–1.00]	2/5 [0.12–0.77]	Yes	8/8 [0.68–1.00]	0.82
Brain Atlas	107/126 [0.78–0.90]	10/10 [0.72–1.00]	14/14 [0.78–1.00]	Yes	22/22 [0.86–1.00]	0.94
Pooled	716/823 [0.85–0.89]	83/83 [0.96–1.00]	45/54 [0.71–0.91]	6/6	111/111 [0.97–1.00]	—
Overall (Gene + Path. + Int.): 844/960 recovered = 0.879, 95% CI [0.857–0.898].						

k: recovered reference items; n: total reference items evaluated. Confidence intervals are 95% Wilson score intervals. The pooled row sums k and n across all six datasets for each metric. Proportion consistency is binary per dataset (Yes/No), summarised as datasets passing (6/6); it has no k/n. Theme coverage counts here are the programmatic keyword-presence metric (k/n); the value in Table 3 of the main text is the independent domain-expert theme score, which is not a k/n proportion and may differ.

S6 Discovery-mode report generation

In discovery mode, ELISA generates an open-ended biological interpretation of a dataset without a predefined target answer. For each query, the retrieval engine returns the top-ranked clusters together with their gene-level evidence (differential expression scores, marker overlaps) and the outputs of the analytical modules (pathway scoring, ligand–receptor interaction inference, and, where applicable, proportional and comparative analyses). This structured evidence is serialized into a prompt and passed to the language model, which produces a narrative report summarizing the cell types, marker genes, pathways, and interactions implicated by the data. In discovery mode, the language model receives only ELISA's data-derived evidence for the queried dataset the retrieved clusters, their gene-level scores, and the outputs of the analytical modules and is not provided with the corresponding reference publication, its results, or its conclusions. The reference publications were used solely to construct the benchmark queries and the expected-cluster ground truth for the retrieval evaluation; they played no role in the generation of the discovery-mode interpretations assessed in Table 4 of the main text. Because the language model is queried

through a frozen API, it retains general biological knowledge from pre-training, but every claim in a discovery-mode report is grounded in the transcriptomic evidence retrieved for that specific dataset rather than in the source publication.

S7 Replication evaluation metrics

Table 3 of the main text reports six metrics quantifying the agreement between ELISA-generated reports and the findings of the corresponding reference publications. Each metric is defined below.

Gene coverage. Gene coverage measures the fraction of key genes highlighted in the reference publication in which ELISA was identified in the correct cell type context. For each dataset, the evaluator compiled a set of key genes from the paper's main text, figures, and supplementary tables (e.g., differentially expressed genes, cell type markers and signaling molecules). A gene was scored as "recovered" if it appeared in ELISA's output for a biologically appropriate cluster. The gene coverage is computed as:

$$\text{Gene coverage} = \frac{|\text{key genes recovered by ELISA}|}{|\text{key genes reported in reference}|} \tag{S2}$$

Pathway alignment. Pathway alignment quantifies whether ELISA's pathway scoring module detects the biological programs reported in the reference study. For each dataset, the evaluator identified the pathways discussed in that paper (e.g., IFN-γ signaling, mTOR and ErbB). A pathway was scored as "aligned" if ELISA's module returned it with a positive score in at least one biologically appropriate cluster. Pathway alignment is computed as:

$$\text{Pathway alignment} = \frac{|\text{pathways found by ELISA}|}{|\text{pathways reported in reference}|} \tag{S3}$$

Interaction recovery. Interaction recovery assesses whether ELISA's ligand-receptor prediction module detected the cell-cell communication axes described in the reference publication. For each dataset, the evaluator compiled ground truth interactions from the paper (e.g., HB-EGF/ERBB4 between macrophages and neuroblasts, HLA-E/NKG2A between epithelial and CD8$^+$ T cells). Recovery was scored at the pair level: a ligand–receptor pair was counted as "recovered" if ELISA detected it with a non-zero score, regardless of whether the source–target cell type assignment exactly matched:

$$\text{Interaction recovery} = \frac{|\text{LR pairs detected by ELISA}|}{|\text{LR pairs reported in reference}|} \tag{S4}$$

Proportion consistency. Proportion consistency is a binary (Yes/No) criterion that evaluates whether ELISA's proportion analysis correctly identified the direction of cell type composition changes for datasets with condition contrasts. For each cell type reported in the reference as increased or decreased in the disease or treatment condition, the evaluator checked whether ELISA's fold change pointed in the same direction. A dataset received "Yes" if the majority of reported changes were directionally consistent.

Theme coverage. Theme coverage captures whether ELISA's interpretive summary reproduced the major biological conclusions of the reference study. Unlike gene and pathway-level metrics, which assess individual molecular entities, theme coverage evaluates high-level biological narratives. For each dataset, the evaluator identified the main themes from the paper's abstract and results (e.g., "aberrant adaptive immunity with upregulated IFN-γ signaling" for the CF dataset;

"therapy-induced macrophage polarization toward immunosuppressive phenotypes" for the neuroblastoma dataset). A theme was scored as "covered" if ELISA's LLM-generated interpretation mentioned and correctly described the corresponding biological finding:

$$\text{Theme coverage} = \frac{|\text{themes captured by ELISA}|}{|\text{major themes in reference}|} \tag{S5}$$

Biological evaluation score. The biological evaluation score provides an independent assessment of overall report quality (see Section S9).

Composite score. The composite score summarizes overall replication performance as the unweighted mean of the four continuous metrics:

$$\text{Composite} = \frac{\text{Gene cov.} + \text{Path. align.} + \text{Int. rec.} + \text{Theme cov.}}{4} \tag{S6}$$

Proportion consistency is excluded from the composite average because it is binary rather than continuous, but is reported separately as a quality check.

S8 Retrieval and analytical evaluation metrics

To ensure reproducible and interpretable evaluation of ELISA's retrieval and analytical modules, we defined the full set of metrics used throughout the benchmark (see also the benchmark scripts in the code repository for complete implementations). Retrieval metrics quantify how effectively each mode recovers the expected cell types for a given query, while analytical metrics assess the accuracy of ELISA's downstream biological interpretation modules interaction discovery, pathway enrichment, proportion analysis, and comparative differential expression. An overview of the six evaluation datasets and their properties is provided in Table S5.

S8.1 Retrieval metrics

Each radar plot in Figure 2 of the main text displays six axes corresponding to three retrieval metrics evaluated separately on the two query categories (ontology and expression). The metrics are:

1. **Cluster Recall@k** (two axes per plot: Ont R@k, Exp R@k). This metric measures the fraction of expected cell types that appear within the top-k positions of the ranked retrieval list. The value of k is adapted to each dataset's number of clusters: R@5 and R@10 for large-cluster datasets (CF Airways with 30 clusters, ICB Multi-Cancer with 31, First-Trimester Brain with 28), R@1 and R@2 for small-cluster datasets (Breast Tissue Atlas with 8 clusters, fdAT2 Organoids with 5, High-Risk Neuroblastoma with 11). A Recall@k of 1.0 indicates that all expected clusters were retrieved within the top-k; a value of 0.0 indicates that none were found. Two Recall cutoffs are shown per plot to capture both stringent (lower k) and permissive (higher k) retrieval accuracy.

2. **Mean Reciprocal Rank** (two axes: Ont MRR, Exp MRR). MRR quantifies the rank position of the *first* correctly retrieved cluster. An MRR of 1.0 means the top-ranked result is relevant; 0.5 means the first relevant result appears at rank 2; 0.33 at rank 3, and so on. MRR captures top-of-list precision, which is critical for interactive use where researchers typically inspect only the first few results.

Together, the six axes capture complementary aspects of retrieval quality: Recall@k measures *coverage* (how many expected clusters are found), whereas MRR measures *precision at rank 1* (how quickly the first relevant cluster appears). Evaluating both metrics on ontology queries

(natural-language, concept-level) and expression queries (gene-signature-based) separately reveals modality-specific strengths: a system may excel at one query type while underperforming the other. The radar footprint thus provides an at-a-glance summary of each retrieval mode's overall coverage, precision, and balance across query types. A larger, more symmetric footprint indicates stronger and more balanced retrieval performance.

The retrieval modes compared are: **Random** (grey dotted line); **BM25** (purple dash-dot line), a classical lexical retriever; **CellWhisperer** (pink dashed line), which uses contrastive text transcriptome CLIP embeddings; **ELISA Semantic** (blue), which performs BioBERT-based cosine similarity matching against cluster descriptions enriched with GO and Reactome terms; **ELISA scGPT** (orange), which scores clusters by matching query genes against per-cluster differential expression profiles; and **ELISA Union** (green), which adaptively fuses both ELISA pipelines by routing each query to the better-performing modality and appending unique results from the secondary pipeline.

S8.2 Statistical testing

To assess whether performance differences between retrieval modes are statistically significant across datasets, we employed one-sided paired t-tests (with the alternative hypothesis that ELISA Union outperforms the baseline) and reported Cohen's d as the paired effect size. Because different datasets use different Recall@k cutoffs, individual metric comparisons have varying sample sizes ($n = 3$ to $n = 6$ datasets). To obtain a single omnibus test, we performed a combined permutation test: the sign of the difference (Union minus baseline) was computed for every metric dataset pair simultaneously, and dataset labels were permuted 50,000 times to construct the null distribution of the aggregate advantage. All p-values and effect sizes are reported in Tables S1–S3 and in Table 2 of the main text.

S9 Human evaluation protocol

To obtain the biological evaluation scores shown in Table 3 of the main text, a domain expert with training in molecular biology and single-cell genomics independently reviewed each ELISA-generated report against the corresponding reference publication. The evaluation followed a structured five-step protocol:

1. **Gene verification.** Each gene reported by ELISA as differentially expressed or as a marker of a specific cell type was cross-checked against the main text, figures, and supplementary tables of the reference publications. A gene was scored as "recovered" if it appeared in the paper's reported DE gene lists, marker panels, or figure annotations for the corresponding cell type. The gene coverage score was computed as the fraction of paper-reported key genes that ELISA identified in the correct cluster context.

2. **Pathway assessment.** Each pathway identified by ELISA's pathway scoring module (e.g., "IFN-gamma signaling," "mTOR signaling") was compared against pathway-level findings described in the reference study. A pathway was scored as "aligned" if the reference publication reported activation or enrichment of that pathway in a consistent cell type context. Pathway alignment was computed as the fraction of paper-reported pathways that ELISA correctly detected as active (score > 0) in at least one biologically appropriate cluster.

3. **Interaction validation.** Each ligand-receptor interaction predicted by ELISA was verified against the cell-cell communication analyses reported in the reference publication. Validation was performed at two levels: (i) whether the ligand–receptor pair itself was reported in the paper, regardless of the cell type context (LR recovery rate), and (ii) whether both the pair and the source target cell type assignment matched the paper's findings (full match rate).

4. **Proportion and condition consistency.** For datasets with condition contrasts (e.g., CF vs. healthy), the evaluator verified whether ELISA's proportion analysis correctly identified the direction of cell type composition changes reported in the reference study. Each cell type with a known expected change (increased or decreased in the disease/treatment condition) was checked for directional agreement.

5. **Theme coverage and hypothesis assessment.** The evaluator assessed whether ELISA's interpretive summaries captured the major biological themes and conclusions of the reference study (e.g., "aberrant adaptive immunity with upregulated IFN-γ signaling" for the CF dataset). Additionally, candidate hypotheses generated by ELISA's discovery mode were evaluated for biological plausibility through targeted literature review: the evaluator searched PubMed for prior evidence supporting or contradicting each proposed mechanism (e.g., CALR–LRP1 in macrophage phagocytosis, TRIM-family ligases in surfactant processing). Hypotheses were classified as "plausible" if supporting literature existed, "novel" if no prior reports were found but the mechanism was biologically coherent, or "unsupported" if contradicted by existing evidence.

The composite score for each dataset was computed as the unweighted mean of gene coverage, pathway alignment, interaction recovery, and theme coverage, with proportion consistency treated as a binary (pass/fail) criterion.

S10 Materials: datasets

ELISA was validated on six publicly available scRNA-seq datasets deposited in the CZ CELLxGENE Discover portal, spanning five distinct tissues, four disease contexts, and both case-control and longitudinal experimental designs (Table S5). Datasets were selected to cover a broad range of biological complexity, cell type diversity, and analytical challenges, including inflammatory lung disease, pediatric and adult cancers, immune checkpoint therapy response, and normal tissue homeostasis.

Dataset 1 (D1): cystic fibrosis bronchial epithelium. Berg *et al.* [1] generated the first single-cell transcriptome atlas of the cystic fibrosis (CF) lung comprising both structural and immune cells. Droplet-based scRNA-seq was performed on bronchial wall biopsies from patients with CF ($n = 8$) and healthy controls ($n = 19$) and integrated using the fastMNN batch correction framework with the Human Lung Cell Atlas as reference. The dataset encompasses approximately 96,000 cells across 30 annotated cell types, including epithelial (basal, ciliated, secretory, goblet, ionocyte), immune ($CD8^+$ T cells, $CD4^+$ T cells, B cells, plasma cells, macrophages, monocytes, NK cells, dendritic cells, mast cells), stromal (fibroblasts, pericytes), and endothelial populations. Key findings include dysregulated basal cell function, aberrant adaptive immunity with upregulated IFN-γ signaling, a novel HLA-E/NKG2A immune checkpoint axis, and altered structural–immune cell crosstalk persisting despite CFTR modulator therapy.

Dataset 2 (D2): high-risk neuroblastoma. Yu *et al.* [2] longitudinally profiled 22 patients with high-risk neuroblastoma before and after induction chemotherapy using single-nucleus RNA and ATAC sequencing combined with whole-genome sequencing. The dataset captures profound therapy-induced shifts in tumor and immune cell subpopulations, identifying enhancer-driven transcriptional regulators of neoplastic states (adrenergic, mesenchymal, proliferative) and macrophage polarization toward pro-angiogenic, immunosuppressive phenotypes. A central finding was the validation of the HB-EGF/ERBB4 paracrine signaling axis between macrophages and neoplastic cells promoting tumor growth through ERK signaling induction.

Dataset 3 (D3): immune checkpoint blockade across cancers. Gondal *et al.* [3] compiled and standardized eight scRNA-seq studies from nine cancer types encompassing 223 patients and over 350,000 cancer cells treated with immune checkpoint blockade (ICB). Cancer types include melanoma, basal cell carcinoma, melanoma brain metastases, triple-negative/HER2-positive/ER-positive breast cancer, clear cell renal carcinoma, hepatocellular carcinoma, and intrahepatic

cholangiocarcinoma. The integrated resource enables cross-cancer investigation of cancer cell-specific ICB responses, with annotations of treatment status, response outcome, and malignant vs. non-malignant cell identity.

Dataset 4 (D4): fetal lung AT2 organoids. Lim *et al.* [4] developed expandable alveolar type 2 (AT2) organoids derived from human fetal lungs at 16–22 post-conception weeks (pcw). Single-cell RNA sequencing of four independent organoid lines (passage 11–16) yielded approximately 9.6k cells across eight annotated cell types, including AT2-like, cycling AT2-like, $CXCL^+$ AT2-like, differentiating basal-like, differentiating pulmonary neuroendocrine, intermediate, neuroendocrine progenitor, and ciliated-like populations. The organoids express mature surfactant proteins (SFTPC, SFTPB, SFTPA1) and markers of surfactant processing (LAMP3, ABCA3, NAPSA), and can differentiate into AT1-like cells. A forward genetic screen identified the E3 ligase ITCH as a key effector of SFTPC maturation, with its depletion phenocopying the pathological SFTPC-I73T variant associated with interstitial lung disease.

Dataset 5 (D5): healthy breast tissue. Bhat-Nakshatri *et al.* [5] constructed a single-cell atlas of healthy breast tissues collected from volunteer donors from the Komen Normal Tissue Bank. Using a rapid procurement and processing protocol, the study profiled breast epithelial and stromal cells, identifying 13 epithelial cell clusters with 23 subclusters exhibiting distinct gene expression signatures. Overlap analysis of subcluster-enriched signatures with breast tumor transcriptomes revealed dominant representation of differentiated luminal subcluster signatures in breast cancers, providing insights into putative cells of origin.

Dataset 6 (D6): first-trimester human brain neurodevelopment. Mannens *et al.* [6] generated a high-resolution multiomic atlas of chromatin accessibility and gene expression across the entire developing human brain during the first trimester (6–13 weeks post-conception). Using scATAC-seq and paired multiome (scATAC-seq + scRNA-seq) sequencing, the study profiled 166k nuclei from 76 biological samples dissected into five antero-posterior segments (telencephalon, diencephalon, mesencephalon, metencephalon, and cerebellum), of which 166,785 nuclei included paired gene expression. The atlas defines 135 clusters spanning neurons (GABAergic, glutamatergic, Purkinje, granule), radial glia, glioblasts, oligodendrocyte progenitor cells, fibroblasts, vascular, and immune cell types. Key findings include over 100 cell-type- and region-specific candidate *cis*-regulatory elements, CNN-predicted enhancer syntax for neuronal specification, elucidation of the ESRRB activation mechanism in the Purkinje cell lineage, and linkage of disease-associated GWAS SNPs to specific neuronal subtypes identifying midbrain-derived GABAergic neurons as particularly vulnerable to major depressive disorder-related mutations.

All datasets were downloaded from CZ CELLxGENE Discover (`https://cellxgene.cziscience.com`) in AnnData (.h5ad) format and preprocessed into ELISA's standardized embedding format (.pt files) as described in Section S11.7. Cell type annotations from the original publications were retained without modification. For datasets with condition metadata (D1, D2, D3, D4), condition columns were mapped to ELISA's comparative analysis framework. Dataset D5 was used to evaluate ELISA's performance on a single-condition atlas without disease contrast, testing the system's capacity for cell type identification and pathway characterization in the absence of differential signals.

S11 Methods

S11.1 ELISA: architecture and design principles

ELISA (Embedding-Linked Interactive Single-cell Agent) is an agent-based computational framework for interactive interrogation of single-cell RNA-seq atlases. The system integrates four core modules a hybrid retrieval engine, an analytical suite, a visualization toolkit, and a large language model (LLM) chat interface to enable biologists to query scRNA-seq datasets using natural language, gene signatures, or a combination of both. The architecture follows a modular design in which each component operates on a shared data representation (a serialized PyTorch embed-

Table S5: Summary of scRNA-seq datasets used for ELISA validation. **Approx. cells**: approximate number of cells or nuclei profiled after quality control. **Cell types**: number of annotated major cell types in the original atlas. **Bench. clusters**: number of annotated populations used as retrieval ground truth in the benchmark, which determines the Recall@k cutoffs in Figure 2 of the main text. **Conditions**: experimental groups or treatment arms.

ID	Tissue	Disease context	Reference	Approx. cells	Cell types	Bench. clusters	Conditions
D1	Lung (bronchial)	Cystic fibrosis	Berg *et al.* [1]	∼96k	30	30	CF vs. Ctrl
D2	Adrenal / tumor	Neuroblastoma	Yu *et al.* [2]	∼372k	20+	11	Pre- vs. post-chemo
D3	Multi-cancer	ICB response	Gondal *et al.* [3]	∼356k	25+	31	R vs. NR; 9 cancers
D4	Lung (fetal)	AT2 organoid model	Lim *et al.* [4]	∼9.6k	8	5	fdAT2 organoid lines
D5	Breast	Healthy tissue atlas	Bhat-Nakshatri *et al.* [5]	∼51k	13	8	Healthy only
D6	Brain (whole)	Neurodevelopment	Mannens *et al.* [6]	∼166k	160	28	6–13 PCW; 5 regions

ding file) and communicates through standardized data structures, enabling extensibility to new datasets without retraining.

The system was implemented in Python 3.10+ and evaluated on six datasets taken from CZ CELLxGENE Discover. All source code, benchmark queries, and evaluation scripts are provided in the accompanying repository.

S11.2 Hybrid retrieval engine

S11.2.1 Query classification and routing

A central design challenge in single-cell atlas retrieval is that user queries span a spectrum from pure natural language ("macrophage infiltration in CF airways") to pure gene signatures ("MARCO FABP4 APOC1 C1QB") and mixed queries combining both. ELISA addresses this through an explicit query classification module that routes each query to the optimal retrieval pipeline.

The classifier operates by tokenizing the input and scoring each token against three criteria: (i) whether it matches a gene name pattern (uppercase alphanumeric, 2–15 characters, with optional hyphenated suffix), (ii) whether it appears in the dataset's known gene vocabulary, and (iii) whether it belongs to a curated set of natural language indicator terms (e.g., "cell", "activation", "signaling"). Queries where $\geq$60% of tokens are classified as gene symbols are routed to the gene pipeline; queries where $\geq$20% of tokens are genes and $\geq$20% are natural language terms are routed to the mixed pipeline; all other queries are routed to the semantic (ontology) pipeline.

S11.2.2 Gene marker scoring pipeline

For gene-list queries, ELISA scores each cluster by evaluating how well its differential expression (DE) profile matches the query genes. For each query gene g found in cluster c's DE statistics, a per-gene score is computed as:

$$\mathrm{score}(g,c) = \big(0.5 + |\log_2 \mathrm{FC}|\big) \times \big(0.3 + \max(\mathrm{pct}_{\mathrm{in}} - \mathrm{pct}_{\mathrm{out}},\ 0)\big) \tag{S7}$$

where $\log_2 \mathrm{FC}$ is the log-fold change of gene g in cluster c, and $\mathrm{pct}_{\mathrm{in}}$ and $\mathrm{pct}_{\mathrm{out}}$ represent the fraction of cells expressing the gene inside and outside the cluster, respectively. The specificity term $(\mathrm{pct}_{\mathrm{in}} - \mathrm{pct}_{\mathrm{out}})$ rewards genes that are selectively enriched in the cluster rather than ubiquitously

expressed. A multiplicative bonus of 1.3× is applied when $\text{pct}_{\text{in}} > 0.5$. The aggregate cluster score is the sum of per-gene scores, modulated by a coverage factor $(0.5 + 0.5 \times n_{\text{found}}/n_{\text{query}})$ that rewards clusters matching more query genes. Three scoring modes are available: 'simple' (binary hit counting), 'weighted' (described above), and 'full' (incorporating adjusted p-value significance via $-\log_{10}(p_{\text{adj}})$, capped at 10).

S11.2.3 Semantic matching pipeline

For ontology and text-based queries, ELISA employs BioBERT [12] (pritamdeka/BioBERT-mnli-snli-scinli-scitail-mednli-stsb) to encode both query text and precomputed cluster descriptions into a shared embedding space. Each cluster's description is constructed during dataset preparation by concatenating its Cell Ontology name, top marker genes (ranked by $|\log_2 \text{FC}|$), enriched Gene Ontology terms, and Reactome pathway annotations, producing a dual-representation embedding that captures both identity and functional context.

At query time, the input text is encoded with BioBERT and cosine similarity is computed against all cluster embeddings. Two augmentation strategies improve retrieval accuracy. First, a name-boosting mechanism adds a score bonus ($\alpha = 0.15$, scaled by word-overlap ratio) when significant substrings (≥4 characters) of a cluster's name appear in the query. Second, a synonym expansion module maps common cell type aliases (e.g., "endothelial" → "endocardial cell"; "NK" → "natural killer cell") to their Cell Ontology equivalents and applies a score boost ($\beta = 0.10$) to matching clusters, addressing vocabulary gaps between colloquial and formal ontology terminology.

S11.2.4 Reciprocal rank fusion for mixed queries

Mixed queries containing both gene names and biological text are handled through reciprocal rank fusion (RRF). Both the gene and semantic pipelines are executed independently, and their ranked outputs are combined using:

$$\text{RRF}(d) = \sum_r \frac{w_r}{k + \text{rank}_r(d) + 1} \tag{S8}$$

where $k = 60$ is the RRF constant, w_r are per-pipeline weights (default: 1.0 for both), and $\text{rank}_r(d)$ is the 0-indexed rank of cluster d in pipeline r. For gene-dominated queries routed through the gene pipeline, a light fusion with the semantic pipeline at a 3:1 weight ratio is applied as a safety mechanism to capture semantically related clusters that lack direct marker gene overlap.

S11.2.5 Additive union evaluation strategy

For benchmarking, we introduce an additive union strategy that maximizes complementarity between modalities. For each query, the modality achieving higher recall@5 against expected clusters is designated as the primary pipeline. The union output begins with the primary pipeline's full ranked list, followed by unique clusters from the secondary pipeline appended in their original rank order. This produces an untruncated result list (up to 2× top-k), evaluated at recall@5, @10, @15, and @20. Ties at recall@5 are broken by mean reciprocal rank (MRR).

S11.3 Analytical modules

S11.3.1 Cell–cell interaction prediction

ELISA predicts ligand–receptor (LR) interactions between cell types using a curated database of 280+ LR pairs spanning 25 signaling pathway categories. The database was compiled from established resources (CellChat [13], CellPhoneDB [14], NicheNet [15]) and augmented with context-specific pairs for cystic fibrosis, neurodegeneration, neuroblastoma, and immune checkpoint biology. Each interaction is represented as a (ligand, receptor, pathway) tuple.

For each source–target cluster pair, the interaction score is computed as:

$$s_{ij} = \text{pct}_{\text{in}}(\text{ligand}, c_i) \times \text{pct}_{\text{in}}(\text{receptor}, c_j) \tag{S9}$$

where $\mathrm{pct_{in}}$ denotes the fraction of cells expressing the gene above detection threshold. Interactions are filtered by minimum expression thresholds (ligand $\geq 10\%$, receptor $\geq 5\%$ by default) and ranked by score. The module outputs per-interaction statistics, pathway-level summaries, and directional pair summaries.

S11.3.2 Pathway activity scoring

Pathway activity across clusters is quantified using curated gene sets encompassing 60+ pathways organized into five categories: immune signaling (IFN-γ, Type I IFN, TNF/NF-κB, JAK-STAT, complement, TLR, chemokine), cell biology (mTOR, PI3K-Akt, Wnt, Notch, Hippo, Hedgehog, cell cycle, apoptosis), neuroscience (glutamatergic/GABAergic synapse, neurodegeneration, FCD progenitor markers), metabolism (oxidative phosphorylation, glycolysis, lipid metabolism, fatty acid metabolism), and tissue-specific programs (surfactant metabolism, epithelial defense, fibrosis, angiogenesis).

For each pathway–cluster combination, the score is computed as the mean $\mathrm{pct_{in}}$ (or alternative metric: $\log_2 \mathrm{FC}$, $\mathrm{pct_{out}}$) across pathway genes detected in the cluster's DE profile, requiring a minimum of 3 genes for a non-zero score. Coverage (fraction of pathway genes detected) is reported alongside scores. Pathway query matching uses word-overlap fuzzy matching to accommodate variant pathway names.

S11.3.3 Comparative analysis

When dataset metadata includes a condition column (e.g., "patient_group" with values "CF" and "Ctrl"), ELISA enables condition-stratified analysis. The module detects condition columns through keyword matching against a curated list (patient_group, condition, disease, treatment, genotype, *etc.*) and validates that the column contains 2–10 distinct values. For each cluster, the condition distribution is estimated from metadata field weights, and a condition bias label is assigned (>60% of cells from one condition). Per-gene statistics ($\log_2 \mathrm{FC}$, $\mathrm{pct_{in}}$, $\mathrm{pct_{out}}$) are reported within condition-biased clusters, and condition-enriched gene lists are compiled across all clusters.

S11.3.4 Proportion analysis

The cell type proportion analysis computes the per-cluster cell counts and fractions relative to the total dataset size. When a condition column is available, the module additionally computes the condition-specific proportions and fold changes. For binary conditions (e.g., CF vs. Control), fold changes were calculated as the fraction of condition A cells in a cluster divided by the fraction of condition B cells, enabling the identification of cell types enriched or depleted in disease states.

S11.3.5 Additional analytical functions

Supplementary analytical functions include: (i) marker specificity scoring, which ranks genes by a weighted score combining specificity $\left(\mathrm{pct_{in}}/(\mathrm{pct_{in}} + \mathrm{pct_{out}})\right)$ and effect size ($|\log_2 \mathrm{FC}|$); (ii) co-expression analysis, computing Pearson correlations of $\mathrm{pct_{in}}$ profiles across clusters; (iii) cell cycle scoring using established S-phase and G2M-phase gene signatures (43 and 54 genes, respectively); and (iv) gene set enrichment against 10 MSigDB Hallmark gene sets.

S11.4 Visualization module

ELISA includes a comprehensive visualization module that generates publication-quality figures in two categories. Retrieval-level visualizations include: embedding landscape projections (UMAP [10], t-SNE [16], or PCA fallback) of cluster-level semantic and expression embeddings, with optional highlighting of retrieved clusters; inter-cluster cosine similarity heatmaps; retrieval score waterfall plots; gene evidence bar charts ($\log_2 \mathrm{FC}$ or $\mathrm{pct_{in}}$); gene-by-cluster heatmaps; radar charts for

multi-metric cluster profiles; semantic vs. expression similarity scatter plots for hybrid retrieval diagnostics; and lambda sweep curves for fusion weight optimization.

When an AnnData (.h5ad) file is provided, cell-level visualizations are generated in a style consistent with Nature and Cell journals: cell-level UMAP plots with Cell Ontology labels placed using a centroid-offset algorithm with iterative repulsion to minimize label overlap; single-gene expression UMAPs with non-expressing cells shown in grey and expression on a purple gradient (capped at the 98th percentile); multi-gene expression grids; and dot plots showing percentage expression (dot size) and z-scored mean expression (dot color) across clusters. All plots used a 40-color colorblind-friendly palette and rasterized cell-level rendering for efficient file sizes.

S11.5 LLM-mediated chat interface

The interactive chat interface wraps all modules behind a command-driven interface that routes user queries to the appropriate pipeline and generates LLM-interpreted summaries. The interface supports six retrieval and analysis modes (semantic, hybrid, discovery, compare, interactions, pathway, proportions) and 15 visualization commands. Each analysis result is automatically accumulated into a session-level report builder.

LLM interpretation is performed via the Groq API using the LLaMA-3.1-8B-Instant model [17] at temperature 0.2. Prompts are constructed with mode-specific templates that enforce strict grounding in dataset evidence: the LLM receives only the retrieved cluster data, gene statistics, and pathway/interaction results as context, with explicit instructions to avoid hallucination, external literature, and causal claims. Context payloads are trimmed to fit within the model's token limits ($\sim$4,500 tokens for user content), with priority given to top-ranked clusters and highest-effect-size genes.

A discovery mode extends standard retrieval by prompting the LLM to produce four structured sections: (i) dataset evidence, (ii) established biology, (iii) consistency analysis identifying matches and mismatches with known biology, and (iv) candidate novel hypotheses stated with probabilistic language. This mode is designed to surface unexpected findings that may represent context-shifted gene functions or novel cell–cell interactions.

S11.6 Benchmarking framework

S11.6.1 Query design

The benchmark comprises 100 queries per dataset divided into two categories: 50 ontology queries (concept-level, testing semantic understanding) and 50 expression queries (gene-signature-based, testing transcriptomic matching). Queries were derived from the findings of the corresponding reference publications, covering the major cell types identified in each study. Each query has a curated set of expected clusters and expected genes, enabling evaluation at both the cluster retrieval and gene delivery levels. The full query sets are listed in Section S14.

S11.6.2 Baseline comparisons

ELISA's retrieval performance was evaluated against CellWhisperer, BM25, and a random ranking, and internally against its Semantic, scGPT, and Additive Union modes.

S11.6.3 Metrics

Retrieval performance was assessed using three metrics. **Cluster Recall@k** measures the fraction of expected clusters appearing in the top-k retrieved results, using fuzzy matching (substring containment or word-overlap Jaccard similarity ≥ 0.5) to accommodate Cell Ontology naming variations. **Mean Reciprocal Rank (MRR)** captures the rank position of the first relevant cluster. **Gene Recall** measures the fraction of expected genes recoverable from the DE profiles

of the top-5 retrieved clusters, assessing whether retrieved clusters collectively provide the gene evidence needed for biological interpretation.

S11.6.4 Analytical module evaluation

Analytical modules were evaluated against ground truth derived from the source publication. Interaction prediction was assessed by ligand–receptor pair recovery rate (whether the correct LR pair was detected regardless of cell type) and full match rate (correct LR pair between the correct source and target cell types, using fuzzy cell type matching). Pathway scoring was evaluated by alignment: the fraction of pathway activities reported in the paper that ELISA correctly identified as active (score > 0) in at least one group. The proportion analysis was evaluated by the consistency rate whether cell types reported as increased or decreased in the disease condition show fold changes in the expected direction. Comparative analysis was evaluated by gene recall, the fraction of differentially expressed genes reported in the paper that can be recovered from ELISA's condition-stratified analysis.

S11.7 Data representation and preprocessing

Each dataset is preprocessed into a single serialized PyTorch file (`.pt`) containing: cluster identifiers, precomputed BioBERT semantic embeddings (768-dimensional, L2-normalized), optional scGPT expression embeddings, per-cluster DE gene statistics ($\log_2$ FC, pct_{in}, pct_{out}, adjusted p-value), per-cluster GO and Reactome enrichment terms, per-cluster metadata (cell counts, condition distributions, categorical field frequencies), cluster text descriptions, and the complete gene vocabulary. This representation enables ELISA to operate entirely at the cluster level without requiring access to the original count matrix, substantially reducing memory requirements and enabling deployment on standard hardware.

S11.8 Software dependencies and reproducibility

ELISA depends on: PyTorch ($\geq$1.12) for tensor operations and data serialization, NumPy for numerical computation, sentence-transformers [8] for BioBERT encoding, scikit-learn for t-SNE projections, UMAP-learn [10] for UMAP projections, matplotlib for visualization, scanpy [9] for AnnData-backed cell-level plots, SciPy for hierarchical clustering and sparse matrix operations, and the Groq Python SDK for LLM access. All analyses were performed on a standard workstation without GPU requirements for the retrieval and analytical modules; BioBERT [12] encoding benefits from but does not require GPU acceleration.

S12 Extended future directions: generalizability across technologies, modalities, and species

The six benchmark datasets in this study were generated with droplet-based scRNA-seq and are all human, and it is therefore important to delineate the extent to which ELISA generalizes beyond this setting. Architecturally, ELISA operates on cluster-level differential-expression statistics and cluster embeddings rather than on raw count matrices or platform-specific features; the retrieval and analytical modules consume the same standardized per-cluster representation regardless of how the underlying counts were generated. This makes the framework largely agnostic to the capture technology: data from plate-based protocols such as Smart-seq2, or from other droplet platforms, can be processed through the identical preprocessing, clustering, and differential-expression pipeline and yield the same embedding format, provided gene-level expression is available. Spatial transcriptomics is a natural extension along this axis and would additionally enable spatially resolved interaction scoring; we regard it as a priority direction rather than a current capability, since spatial coordinates are not yet consumed by the interaction module.

The principal current limitation to generalization is species rather than technology. ELISA's expression-side representation is produced by the whole-human scGPT foundation model, and its semantic side by BioBERT, a biomedical language model trained predominantly on human-centric literature. Both components therefore assume human gene symbols and human biological context. Applying ELISA to non-human data (for example mouse or zebrafish) would require either mapping the species' genes to their human orthologs, which is a practical stopgap but is incomplete for genes lacking a one-to-one ortholog, or, preferably, substituting a species-appropriate expression foundation model (e.g., a mouse-pretrained scGPT variant) together with a text encoder whose training covers the corresponding non-human literature. Because ELISA treats the expression foundation model and the semantic encoder as interchangeable, frozen components behind a fixed interface, such substitution is a natural and self-contained extension that does not require redesigning the retrieval, analytical, or interpretation layers. Systematic cross-species benchmarking, including the construction of species-specific reference ground truth analogous to the human benchmark used here, is an important direction for future work.

S13 ELISA parameters and hyperparameters

Tables S6–S9 report all parameters and hyperparameters used in the ELISA framework. Default values were used throughout all experiments; no dataset-specific tuning was performed.

Table S6: Data preprocessing and embedding generation parameters.

Parameter	Value	Description
Preprocessing (Scanpy)		
`target_sum`	10,000	Library-size normalization target
`n_top_genes`	3,000	HVGs selected (Seurat v3)
`max_value`	10	Z-score clipping threshold
`n_comps`	50	PCA components
Leiden resolution	1.0	Used only if no annotations exist
Differential expression		
Method	Wilcoxon	Via `scanpy.tl.rank_genes_groups`
`DE_PVAL`	0.10	Adjusted p-value cutoff
`TOP_K_MARKERS_STATS`	10,000	Max genes stored per cluster
`TOP_K_MARKERS_TEXT`	400	Genes in cluster text summaries
Enrichment (gseapy)		
Gene sets	GO_Biological_Process_2023, Reactome_2022	
`TOP_K_GO`	15	GO terms retained per cluster
`TOP_K_REACTOME`	15	Reactome terms per cluster
Enrichment cutoff	0.05	Adjusted p-value threshold
Input genes	200	Top DE genes per enrichment call
Semantic embedding (BioBERT)		
Model	`pritamdeka/BioBERT-mnli-snli-scinli-scitail-mednli-stsb`	
Embedding dim	768	Output dimensionality
α (`IDENTITY_ALPHA`)	0.6	Identity vs. context weight
Normalization	L2	Final combined embeddings
Batch size	16	Sentences per encoding batch
scGPT expression embedding		
Model	scGPT whole-human	Pre-trained foundation model
Embedding dim	512	CLS token dimensionality
`N_BINS`	51	Expression binning resolution
`MAX_TOKENS`	3,000	Max gene tokens per cell
Batch size	64	Cells per inference batch
Aggregation	Mean pooling	Cell → cluster centroids
Normalization	L2	Cluster-level centroids

Table S7: Hybrid retrieval engine parameters.

Parameter	Value	Description
Query classification		
Gene threshold	$\geq$60%	Token fraction to route as gene query
Mixed threshold	$\geq$20% each	Gene + NL tokens for mixed routing
Gene pattern	A–Z, 2–15 chars	Regex for gene symbol detection
Gene marker scoring		
Score function	$(0.5 + \lvert \log_2 \mathrm{FC} \rvert) \times (0.3 + \max(\mathrm{pct_{in}} - \mathrm{pct_{out}}, 0))$	
High-expr bonus	$\times 1.3$	When $\mathrm{pct_{in}} > 0.5$
Coverage factor	$0.5 + 0.5 \times n_{\mathrm{found}}/n_{\mathrm{query}}$	
Semantic matching		
Similarity	Cosine	Query vs. cluster embeddings
Name boost (α)	0.15	Bonus for ontology name overlap
Min substring	4 chars	For name boost activation
Synonym boost (β)	0.10	Bonus for synonym match
Reciprocal rank fusion		
RRF constant (k)	60	Smoothing constant
Weights	1.0 : 1.0	Gene : semantic
Additive union (benchmarking)		
Primary selection	Recall@5	Higher-recall modality is primary
Tiebreaker	MRR	When Recall@5 is tied
Default settings		
`top_k`	5	Clusters returned per query
`pre_k`	40	Candidates before reranking
γ	2.5	Reranking sharpness
λ_{sem} (scGPT)	0.0	Pure gene scoring mode
λ_{sem} (discovery)	0.5	Balanced mode

Table S8: Analytical module parameters.

Parameter	Value	Description
Ligand–receptor interactions		
Database size	280+ pairs	From CellChat, CellPhoneDB, NicheNet
Pathway categories	25	Signaling annotations
`min_ligand_pct`	0.10	Min ligand expr. in source
`min_receptor_pct`	0.05	Min receptor expr. in target
Score	$\mathrm{pct}_{\mathrm{in}}(L) \times \mathrm{pct}_{\mathrm{in}}(R)$	Expression fraction product
Self-interactions	Excluded	Source $\neq$ target
Pathway activity scoring		
Number of pathways	60+	Across 5 categories
Metric	Mean $\mathrm{pct}_{\mathrm{in}}$	Avg. expression of pathway genes
`min_genes`	3	Min for non-zero score
Categories	Immune, Cell biology, Neuroscience, Metabolism, Tissue-specific	
Comparative analysis		
Condition bias	>60%	Fraction to assign bias label
`min_pct`	0.05	Min expr. for gene inclusion
`top_n`	20	Genes per cluster
Enriched genes	30	Per-condition summary limit
Proportion analysis		
Fold change	$\mathrm{frac}_A/\mathrm{frac}_B$	Condition ratio
Min denominator	0.001	Below: reported as ∞
Cell cycle scoring		
S-phase genes	43	Seurat S-phase markers
G2M-phase genes	54	Seurat G2M markers
Cycling threshold	S>0.3 and G2M>0.3	Both above threshold
Gene set enrichment		
Default gene sets	10 MSigDB Hallmark	Curated pathways
`min_genes`	3	Min for non-zero score

Table S9: LLM interpretation parameters.

Parameter	Value	Description
LLM configuration		
Default provider	Groq	Free tier, 500K tokens/day
Default model	LLaMA-3.1-8B	Via Groq Cloud API
Supported	4 providers	Groq, Gemini, OpenAI, Claude
Temperature	0.2	Low for reproducibility
Prompt limit	18,000 chars	≈4,500 tokens
Context limit	12,000 chars	≈3,000 tokens
Safety and rate limiting		
Spending cap	EUR 1.00	Hard cap, configurable
Max retries	5	On rate-limit errors
Initial wait	10 s	Backoff start
Backoff	Exponential	Max 120 s
Context trimming		
Clusters	Top 10	In compare mode
Gene evidence	Top 5	Per cluster
Pathway scores	Top 10	Entries to LLM
Interactions	Top 20	Entries to LLM
Discovery sections	4	Evidence, Biology, Consistency, Hypotheses

S14 Benchmark queries

The complete ontology and expression query sets used in the retrieval benchmark are listed below, grouped by dataset (D1–D6 as defined in Table S5).

S14.1 D1: Cystic Fibrosis Airways (Berg *et al.* [1])

S14.1.1 Ontology queries

Q1. Macrophage and monocyte infiltration in cystic fibrosis airways

Q2. Recruited monocytes and pro-inflammatory macrophages in CF lung tissue

Q3. Macrophage scavenging receptor expression and phagocytosis in CF

Q4. Non-classical monocyte patrol function in CF bronchial wall

Q5. CD8 T cell activation and cytotoxicity in CF lung inflammation

Q6. CD8 T cell inflammatory cytokine production and IFNG signaling in CF

Q7. HLA-E CD94 NKG2A immune checkpoint inhibiting CD8 T cell activity

Q8. Dysfunctional CD8 T cell response to chronic *Pseudomonas* infection in CF

Q9. CALR LRP1 interaction between T cells and macrophages promoting inflammation

Q10. CD4 helper T cell immune activation in cystic fibrosis

Q11. CD4 T cell VEGF receptor signaling and hypoxia response in CF

Q12. Aberrant Th2 and Th17 T cell responses in *Pseudomonas*-infected CF lungs

Q13. Chronic adaptive immune activation of T lymphocytes in CF despite modulator therapy

Q14. B cell activation and immunoglobulin response in CF airways

Q15. B cell receptor downregulation and reduced plasma cell markers in CF

Q16. Interferon gamma signaling and HLA-DP expression in B cells of CF patients

Q17. PDGFRB signaling pathway activated in B cells from CF lungs

Q18. Basal cell dysfunction and reduced stemness in cystic fibrosis epithelium

Q19. Impaired basal cell differentiation and pathogenic basal cell variants in CF

Q20. Basal cell DNA damage repair and chromatin remodeling in CF airways

Q21. Reduced keratinization gene expression CSTA HSPB1 in CF basal cells

Q22. Basal cell altered cell–cell communication and increased interactions in CF

Q23. Ciliated cell ciliogenesis and increased abundance in CF bronchial epithelium

Q24. Ciliated cell HLA class II expression and immune-linked transcriptional changes in CF

Q25. Skewed basal cell differentiation towards ciliated cells in CF epithelium

Q26. Natural killer cell cytotoxicity and NKG2A immune checkpoint in CF

Q27. NKG2A blockade to restore NK and CD8 T cell function in CF lung

Q28. Innate lymphoid cell dysfunction and impaired antimicrobial defense in CF

Q29. Pulmonary ionocyte CFTR expression in cystic fibrosis

Q30. Ionocyte unique cell–cell interactions with adaptive lymphocytes in CF

Q31. Endothelial cell remodeling and VEGF signaling in CF lung

Q32. Reduced endothelial cell proportions and altered differentiation in CF airways

Q33. Hypoxia-induced VEGF upregulation and vascular remodeling in CF lungs

Q34. Dendritic cell antigen presentation in CF airways

Q35. IFNG IFNGR2 interaction between CD8 T cells and dendritic cells in CF

Q36. Mast cell degranulation and allergic inflammation in CF

Q37. Secretory cell mucus overproduction and inflammatory signaling in CF epithelium

Q38. Goblet cell hyperplasia and mucin gene expression in cystic fibrosis

Q39. Submucosal gland epithelial cell changes in cystic fibrosis

Q40. Reduced submucosal gland cell proportions and gland development dysfunction in CF

Q41. Type I interferon response and inflammatory signaling in CF epithelial cells

Q42. Interferon responsive gene upregulation across epithelial subsets in CF

Q43. VEGF receptor signaling and hypoxia response across cell types in CF

Q44. TXNIP-mediated NLRP3 inflammasome activation in CF lymphocytes and epithelial cells

Q45. GNAI2 immunomodulatory signaling in CD8 T cells and B cells in CF

Q46. GNAI2 adenylate cyclase regulation and CFTR function in lymphocytes

Q47. Stromal cell and fibroblast remodeling in CF airway tissue

Q48. Pericyte and stromal cell contribution to airway fibrosis in CF

Q49. IFNG–IFNGR1 interaction between CD8 T cells and basal cells, macrophages, and endothelial cells in CF

Q50. Altered structural–immune cell crosstalk in CF involving lymphocytes, ionocytes, and macrophages

S14.1.2 Expression queries

Q51. `MARCO FABP4 APOC1 C1QB C1QC MSR1`

Q52. `CD68 CD14 CSF1R CSF2RA LGALS2`

Q53. `G0S2 FABP4 PPARG APOC1 C1QB`

Q54. `FCGR3A CX3CR1 CD14 CDKN1C LILRB2`

Q55. `CD8A CD8B GZMB PRF1 IFNG NKG7`

Q56. `IFNG GNAI2 CD69 CD81 CD3G FOS JUND`

Q57. `GZMB PRF1 NKG7 GNLY KLRD1 CD8A`

Q58. TXNIP MAP2K2 IFNG CD81 CD3G CD69

Q59. KLF2 IL7R CD48 TXNIP ETS1

Q60. CD3D CD4 IL7R CD3E CD3G

Q61. TRAJ52 TRBV22-1 TRDJ2 CD3E CD3G

Q62. CD3G CD3E CD69 IL7R CD81 FOS

Q63. IGLJ3 IGKJ1 IGHJ5 JCHAIN MZB1 XBP1

Q64. CD79A IGHG3 IGLC2 SYK CD81 JCHAIN

Q65. SYK CSK CD9 CD81 JUND LTB HLA-DPA1

Q66. IGHG3 IGLC2 IGHD IGHA1 IGLC1 IGLC3

Q67. KRT5 KRT14 KRT15 TP63 IL33 CSTA

Q68. CSTA HSPB1 KRT5 KRT14 TP63

Q69. KRT5 IL33 TP63 KRT15 LAMB3 COL17A1

Q70. FOXJ1 DNAH5 CAPS PIFO RSPH1 DNAI1

Q71. DNAH5 SYNE1 SYNE2 CAPS PIFO

Q72. GNLY KLRD1 KLRK1 NKG7 PRF1 GZMB

Q73. GNLY NKG7 KLRD1 KLRK1 KLRC1

Q74. ATP6V1G3 FOXI1 BSND CLCNKB ASCL3

Q75. FOXI1 CFTR ATP6V1G3 BSND RARRES2

Q76. PLVAP ACKR1 ERG VWF PECAM1 CDH5

Q77. VIM PLVAP ACKR1 MGP PTGDS CXCL14

Q78. CPA3 TPSAB1 TPSB2 MS4A2 HDC GATA2

Q79. TPSAB1 TPSB2 KIT CPA3 MS4A2

Q80. HLA-DPA1 HLA-DRB1 CD74 GPR183 LGALS2

Q81. HLA-DPA1 HLA-DPB1 HLA-DRB1 CD80 CD86 CD74

Q82. SCGB1A1 SCGB3A1 MUC5AC MUC5B LYPD2 PRR4

Q83. SCGB1A1 MUC5AC SCGB3A1 LYPD2

Q84. MUC5AC MUC5B LYZ SCGB1A1 SCGB3A1

Q85. COL1A2 LUM DCN SFRP2 COL3A1 PDGFRA

Q86. PDGFRA COL1A2 COL3A1 VCAN DCN LUM

Q87. PDGFRB VIM COL1A2 MGP CXCL14

Q88. SST CHGA ASCL1 GRP CALCA SYP

Q89. GRP ASCL1 SYT1 CHGA SYP CALCA

Q90. `HLA-E KLRC1 KLRD1 KLRC2 KLRC3 KLRK1`

Q91. `HLA-E KLRC1 KLRD1 CD8A CD8B`

Q92. `CALR LRP1 GNAI2 FOS JUND MAP2K2`

Q93. `GNAI2 CXCR3 F2R S1PR4 CD69`

Q94. `IFIT1 MX1 OAS2 ISG15 IFITM3 IFIT3`

Q95. `IFIT1 MX1 OAS2 IFIT3 IFI6`

Q96. `KDM1A KMT5A RAD50 ERCC6 ERCC8`

Q97. `TXNIP MAP2K2 ETS1 VEGFA KLF2`

Q98. `IFNG IFNGR1 IFNGR2 CALR LRP1`

Q99. `CCL5 CCR5 CXCL10 CXCR3 F2R`

Q100. `CFTR FOXI1 SCGB1A1 KRT5 FOXJ1 MUC5AC`

S14.2 D2: High-Risk Neuroblastoma (Yu *et al.* [2])

S14.2.1 Ontology queries

Q1. Neuroblast neoplastic cell of sympathetic nervous system expressing PHOX2B and ISL1

Q2. Neuroblastoma tumor cell with MYCN amplification and proliferative phenotype

Q3. Adrenergic neuroblast expressing catecholamine biosynthesis enzymes tyrosine hydroxylase

Q4. Neuroblastoma cell with calcium and synaptic signaling pathway enrichment

Q5. Dopaminergic neuroblast expressing dopamine transporter and metabolic genes

Q6. Proliferating neuroblastoma cell with cell cycle and DNA replication markers

Q7. Mesenchymal neuroblastoma cell state expressing extracellular matrix genes and YAP1

Q8. Intermediate OXPHOS neuroblast with ribosomal gene expression and oxidative phosphorylation

Q9. EZH2 expressing neuroblastoma cell PRC2 polycomb repressive complex chromatin regulation

Q10. Neuroblastoma cell ERBB4 receptor expressing epidermal growth factor signaling

Q11. Neuroblast with adrenergic transcription factor PHOX2A PHOX2B GATA3 expression

Q12. Neural crest derived neoplastic cell in pediatric tumor expressing chromogranin

Q13. Neuroblastoma cell immune evasion NECTIN2 and checkpoint ligand expression

Q14. Mesenchymal transition state in neuroblastoma with AP-1 transcription factors

Q15. Tumor associated macrophage in neuroblastoma microenvironment CD68 CD163 expressing

Q16. Pro-inflammatory macrophage IL18 expressing anti-tumor immune response

Q17. Pro-angiogenic macrophage VCAN expressing promoting tumor vascularization

Q18. Immunosuppressive macrophage C1QC SPP1 complement expressing in tumor

Q19. Tissue resident macrophage F13A1 expressing phagocytic function in neuroblastoma

Q20. Lipid associated macrophage HS3ST2 with metabolic phenotype in tumor

Q21. Macrophage secreting HB-EGF ligand for ERBB4 receptor activation on neuroblasts

Q22. CCL4 expressing pro-angiogenic macrophage chemokine signaling in tumor

Q23. Proliferating macrophage MKI67 TOP2A expanding after chemotherapy

Q24. THY1 positive macrophage undefined myeloid phenotype in neuroblastoma

Q25. T cell lymphocyte infiltrating neuroblastoma tumor expressing CD247 CD96

Q26. Cytotoxic T cell with granzyme perforin mediated tumor cell killing

Q27. Tumor infiltrating T lymphocyte immune response to neuroblastoma

Q28. B cell lymphocyte PAX5 MS4A1 in neuroblastoma tumor immune microenvironment

Q29. B lymphocyte humoral immunity and antigen presentation in pediatric tumor

Q30. Dendritic cell IRF8 FLT3 antigen presentation priming T cell responses in tumor

Q31. Professional antigen presenting dendritic cell MHC class II expression

Q32. Fibroblast stromal cell PDGFRB DCN extracellular matrix production in neuroblastoma

Q33. Cancer associated fibroblast FAP ACTA2 expressing in tumor stroma

Q34. Neural crest derived endoneurial fibroblast in neuroblastoma tissue

Q35. Schwann cell PLP1 CDH19 myelinating glial cell in neuroblastoma microenvironment

Q36. Schwann cell precursor neural crest lineage expanding after therapy

Q37. Endothelial cell PECAM1 PTPRB vascular marker in neuroblastoma tumor vasculature

Q38. Tumor endothelium blood vessel lining cell expressing vascular endothelial markers

Q39. Adrenal cortex cell steroidogenesis CYP11A1 CYP11B1 adjacent normal tissue

Q40. Cortical cell of adrenal gland steroid hormone biosynthesis normal adjacent tissue

Q41. Hepatocyte ALB expressing liver cell from adjacent normal tissue in neuroblastoma biopsy

Q42. Kidney cell renal tissue PKHD1 from adjacent normal tissue in neuroblastoma specimen

Q43. Chemotherapy induced tumor microenvironment rewiring macrophage expansion after therapy

Q44. HB-EGF ERBB4 paracrine signaling axis between macrophage and neuroblast promoting ERK

Q45. Tumor immune evasion and antigen presentation in neuroblastoma

Q46. VEGFA angiogenesis signaling in neuroblastoma tumor microenvironment

Q47. Immune cell infiltration in high-risk neuroblastoma T cell B cell macrophage

Q48. THBS1 CD47 don't eat me signal between macrophage and neuroblastoma cell

Q49. Neuroblastoma cell expressing ALK receptor tyrosine kinase oncogenic driver

Q50. Tumor microenvironment cell diversity neuroblasts fibroblasts Schwann endothelial macrophages

S14.2.2 Expression queries

Q51. PHOX2B ISL1 HAND2 TH DBH DDC CHGA

Q52. MYCN MKI67 TOP2A EZH2 SMC4 BIRC5

Q53. PHOX2A PHOX2B GATA3 ASCL1 ISL1 HAND2

Q54. CACNA1B SYN2 KCNMA1 KCNQ3 GPC5 CREB5

Q55. SLC18A2 TH DDC AGTR2 ATP2A2 PHOX2B

Q56. MKI67 TOP2A EZH2 SMC4 BIRC5 BUB1B ASPM KIF11

Q57. YAP1 FN1 VIM COL1A1 SERPINE1 SPARC THBS2

Q58. ERBB4 EGFR HBEGF TGFA EREG AREG

Q59. NECTIN2 CD274 B2M HLA-A HLA-B PHOX2B

Q60. JUN FOS JUNB JUND FOSL2 BACH1 BACH2

Q61. CHGA CHGB PHOX2B ISL1 NTRK1 RET

Q62. ETS1 ETV6 ELF1 KLF6 KLF7 RUNX1 ZNF148

Q63. ALK MYCN NTRK2 PHOX2B TH

Q64. CD68 CD163 CD86 CSF1R MRC1 SPP1

Q65. IL18 CD68 CD163 CD86 HLA-DRA CSF1R

Q66. VCAN VEGFA CD68 CD163 SPP1 EGFR

Q67. C1QC SPP1 CD68 CD163 APOE TREM2

Q68. F13A1 CD68 CD163 MRC1 LYVE1 CSF1R

Q69. HS3ST2 CYP27A1 CD68 CD163 APOE LPL

Q70. HBEGF TGFA EREG AREG CD68 CD163

Q71. CCL4 CD68 CD163 VEGFA CSF1R CCL3

Q72. THY1 CD68 CD163 MRC1 CSF1R CD86

Q73. CD247 CD96 CD3D CD3E CD8A CD4

Q74. GZMA GZMB PRF1 IFNG CD8A CD3D

Q75. PAX5 MS4A1 CD19 CD79A HLA-DRA HLA-DRB1

Q76. IRF8 FLT3 CLEC9A CD1C CD80 HLA-DRA

Q77. PDGFRB DCN LUM COL1A1 COL1A2 VIM

Q78. FAP ACTA2 COL1A1 PDGFRA DCN LUM

Q79. PLP1 CDH19 SOX10 MPZ MBP S100B

Q80. PECAM1 PTPRB CDH5 VWF KDR FLT1

Q81. CYP11A1 CYP11B1 CYP17A1 STAR NR5A1

Q82. `ALB DCDC2 HNF4A APOB`

Q83. `PKHD1 PAX2 WT1 SLC12A1`

Q84. `PHOX2B CD68 CD3D MS4A1 PECAM1 DCN PLP1`

Q85. `HBEGF ERBB4 CD68 PHOX2B MAPK1`

Q86. `VCAN THBS1 CD47 ITGB1 CD68 PHOX2B`

Q87. `HLA-A HLA-B HLA-C B2M HLA-DRA HLA-DRB1`

Q88. `VEGFA KDR FLT1 NRP1 GPC1 PECAM1`

Q89. `CD68 IL18 VCAN C1QC SPP1 F13A1 HS3ST2 CCL4 THY1`

Q90. `PHOX2B MKI67 TOP2A YAP1 CACNA1B SLC18A2`

Q91. `APOE LDLR VLDLR LPL HS3ST2 CD68`

Q92. `THBS1 ITGB1 ITGA3 LRP5 CD47 FN1`

Q93. `COL1A1 COL1A2 COL4A1 COL4A2 FN1 VIM SPARC`

Q94. `MAPK1 MAPK3 AKT1 ERBB4 EGFR HBEGF`

Q95. `CD274 PDCD1 CTLA4 TIGIT LAG3 NECTIN2`

Q96. `PHOX2B CD68 PLP1 PECAM1 DCN IRF8 PAX5 CD247`

Q97. `CYP11A1 ALB PKHD1 PHOX2B CD68`

Q98. `PHOX2B HBEGF ERBB4 VCAN SPP1 CD163 VEGFA`

Q99. `MKI67 TOP2A PCNA CDK1 CCNB1 EZH2 MELK`

Q100. `PHOX2B ISL1 CD68 CD163 CD3D MS4A1 PLP1 PECAM1 DCN CYP11A1 ALB`

S14.3 D3: Immune Checkpoint Blockade Multi-Cancer (Gondal *et al.* [3])

S14.3.1 Ontology queries

Q1. Malignant cancer cell expressing immune checkpoint ligand PD-L1 for immune evasion

Q2. Tumor cell immune evasion through HLA downregulation and B2M loss

Q3. Melanoma cancer cell expressing MITF MLANA PMEL lineage markers

Q4. Breast cancer epithelial cell markers EPCAM KRT8 KRT18 KRT19 in ICB treated tumors

Q5. Tumor cell proliferation and cell cycle markers in malignant cells

Q6. Cancer cell VEGFA and TGFB1 immunosuppressive signaling in tumor microenvironment

Q7. Epithelial mesenchymal transition EMT markers in cancer cells during ICB treatment

Q8. Effector CD8 T cell cytotoxic function with granzyme and perforin expression

Q9. Activated CD8 T cell expressing IFNG and TNF anti-tumor cytokines

Q10. CD8 T cell exhaustion with PD-1 LAG3 TIM3 TIGIT checkpoint receptor co-expression

Q11. TOX transcription factor driving T cell exhaustion program in chronic antigen stimulation

Q12. Central memory CD8 T cell with TCF7 and IL7R expression for long-lived immunity

Q13. Naive CD8 T cell expressing CCR7 SELL before antigen encounter

Q14. CD8-positive T cell co-stimulatory receptor 4-1BB ICOS upon activation

Q15. CD4 positive helper T cell TCR signaling and cytokine production

Q16. Regulatory T cell FOXP3 expressing immunosuppressive function in tumor

Q17. T follicular helper cell CXCR5 BCL6 supporting B cell responses in tertiary lymphoid structures

Q18. Th17 helper T cell IL17A RORC inflammatory response in tumor microenvironment

Q19. CD8-positive CD28-negative regulatory T cell with suppressive function

Q20. Natural killer T cell NKT innate cytotoxicity with KLRD1 and NKG7 expression

Q21. NK cell mediated tumor killing through NCR1 and KLRB1 receptor activation

Q22. B cell CD19 MS4A1 CD79A antigen presentation and humoral immunity in tumor

Q23. Plasma cell antibody secreting immunoglobulin production SDC1 MZB1

Q24. Tertiary lymphoid structure B cell and plasma cell formation in ICB-responsive tumors

Q25. Tumor associated macrophage M2 polarization CD163 MRC1 immunosuppressive function

Q26. Macrophage complement expression C1QA C1QB and TREM2 in tumor microenvironment

Q27. Classical monocyte CD14 LYZ infiltration into tumor during checkpoint blockade

Q28. Dendritic cell antigen presentation CD80 CD86 priming T cell responses

Q29. Plasmacytoid dendritic cell IRF7 LILRA4 type I interferon production

Q30. Myeloid cell general CSF1R ITGAM expressing innate immune population

Q31. Mast cell KIT TPSB2 CPA3 in allergic and inflammatory tumor responses

Q32. Microglial cell brain resident macrophage in melanoma brain metastasis

Q33. Cancer associated fibroblast FAP ACTA2 COL1A1 producing extracellular matrix

Q34. Myofibroblast ACTA2 TAGLN contractile smooth muscle actin expression in tumor stroma

Q35. Tumor endothelial cell PECAM1 CDH5 VWF vascular marker expression

Q36. Melanocyte pigmentation pathway MITF TYR TYRP1 DCT lineage genes

Q37. Hematopoietic multipotent progenitor cell stem cell marker expression

Q38. PD-1 blockade restoring effector CD8 T cell anti-tumor cytotoxicity

Q39. CTLA-4 blockade enhancing CD4 helper T cell and reducing Treg suppression

Q40. T cell clonal replacement and expansion following PD-1 checkpoint inhibition

Q41. TCF4 dependent resistance program in mesenchymal-like melanoma cells

Q42. T cell exclusion program in tumor cells resisting checkpoint blockade therapy

Q43. Antigen processing and MHC class I presentation in tumor cells

Q44. MHC class II antigen presentation by professional antigen presenting cells

Q45. Interferon gamma response driving PD-L1 upregulation on tumor cells

Q46. Tumor infiltrating lymphocyte diversity including T B and NK cells

Q47. Liver cancer hepatocellular carcinoma markers ALB AFP GPC3 in ICB dataset

Q48. Clear cell renal carcinoma CA9 PAX8 markers in kidney cancer patients

Q49. Basal cell carcinoma Hedgehog pathway PTCH1 GLI1 GLI2 SHH signaling

Q50. Lymphocyte general population in tumor immune microenvironment

S14.3.2 Expression queries

Q51. `CD274 PDCD1LG2 B2M HLA-A CD47 IDO1 VEGFA`

Q52. `MITF MLANA PMEL TYR DCT SOX10 TYRP1`

Q53. `EPCAM KRT8 KRT18 KRT19 MUC1 CDH1 ESR1`

Q54. `MKI67 TOP2A PCNA CD274 B2M TGFB1`

Q55. `PRF1 GZMA GZMB GZMK GNLY NKG7 IFNG`

Q56. `GZMB PRF1 IFNG TNF FASLG NKG7 CD8A`

Q57. `CD69 ICOS TNFRSF9 IFNG GZMB CD8A`

Q58. `PDCD1 LAG3 HAVCR2 TIGIT TOX ENTPD1`

Q59. `TOX TOX2 PDCD1 HAVCR2 LAG3 TIGIT BTLA`

Q60. `TCF7 LEF1 CCR7 SELL IL7R CD8A CD8B`

Q61. `CCR7 SELL TCF7 LEF1 IL7R CD3D`

Q62. `CD4 CD3D CD3E IL7R CD28 ICOS TCF7`

Q63. `FOXP3 IL2RA CTLA4 IKZF2 TNFRSF18 TIGIT`

Q64. `CXCR5 BCL6 ICOS PDCD1 CD4 CD3D`

Q65. `RORC IL17A IL23R CCR6 CD4 CD3E`

Q66. `CD8A GZMB PRF1 LAG3 CTLA4 PDCD1`

Q67. `KLRD1 KLRK1 NKG7 GNLY PRF1 GZMB NCAM1`

Q68. `NCAM1 NCR1 KLRB1 KLRC1 GZMB IFNG`

Q69. `CD19 MS4A1 CD79A CD79B HLA-DRA HLA-DRB1`

Q70. `SDC1 MZB1 JCHAIN IGHG1 IGKC CD79A`

Q71. `CD163 MRC1 MSR1 MARCO CD68 APOE TREM2`

Q72. `C1QA C1QB APOE TREM2 CD68 SPP1`

Q73. `CD14 FCGR3A S100A8 S100A9 LYZ CSF1R`

Q74. CD80 CD86 CD83 CCR7 HLA-DRA CLEC9A

Q75. LILRA4 IRF7 IRF8 IL3RA NRP1

Q76. ITGAM CSF1R CD68 LYZ S100A8 S100A9

Q77. KIT TPSB2 TPSAB1 CPA3 HPGDS HDC

Q78. P2RY12 TMEM119 CX3CR1 CSF1R AIF1

Q79. FAP ACTA2 COL1A1 COL1A2 PDGFRA DCN LUM

Q80. ACTA2 TAGLN MYH11 COL1A1 PDGFRB VIM

Q81. PECAM1 CDH5 VWF KDR FLT1 ENG

Q82. MITF TYR TYRP1 DCT MLANA PMEL SOX10

Q83. CD34 KIT FLT3 PROM1 THY1 PTPRC

Q84. CD3D CD3E CD8A CD4 TRAC TRBC1

Q85. HLA-DRA HLA-DRB1 HLA-DPA1 HLA-DPB1 CD74 CIITA

Q86. HLA-A HLA-B HLA-C B2M TAP1 TAP2

Q87. PDCD1 CD274 CTLA4 CD80 CD86 LAG3 HAVCR2

Q88. CD274 CD47 IDO1 GZMB PRF1 IFNG

Q89. CD8A CD4 MS4A1 CD68 PECAM1 FAP EPCAM NCAM1

Q90. GZMB IFNG FOXP3 CD163 CD274 MS4A1 PECAM1

Q91. ALB AFP GPC3 EPCAM KRT19

Q92. CA9 PAX8 MME EPCAM VEGFA

Q93. PTCH1 GLI1 GLI2 EPCAM KRT14

Q94. ERBB2 ESR1 EPCAM KRT8 KRT18 MUC1

Q95. CCR7 SELL TCF7 PDCD1 TOX GZMB PRF1

Q96. IFNG CD274 STAT1 IRF1 B2M HLA-A

Q97. CD8A CD4 FOXP3 CXCR5 RORC CCR7 KLRD1 CD3D

Q98. CD68 CD163 CD14 S100A8 CD80 KIT LILRA4 ITGAM

Q99. FAP ACTA2 PECAM1 CDH5 COL1A1 PDGFRA VWF

Q100. CD274 GZMB CD68 MS4A1 FAP PECAM1 MITF FOXP3 CD8A KIT LILRA4

S14.4 D4: Fetal Lung AT2 Organoids (Lim *et al.* [4])

S14.4.1 Ontology queries

Q1. Alveolar type 2 cell identity and surfactant protein production in fetal lung organoids

Q2. Mature AT2 cell markers and lamellar body formation in fdAT2 organoids

Q3. Surfactant protein C maturation and intracellular trafficking in alveolar epithelium

Q4. SFTPC processing through endosomal compartments and multivesicular bodies

Q5. Surfactant secretion and lamellar body exocytosis in human AT2 cells

Q6. ITCH E3 ubiquitin ligase role in SFTPC trafficking and ubiquitination

Q7. K63 ubiquitination of surfactant protein C for ESCRT recognition and MVB entry

Q8. HECT domain E3 ligase ITCH depletion phenocopying SFTPC-I73T pathogenic variant

Q9. Ubiquitome forward genetic screen for SFTPC trafficking effectors

Q10. SFTPC relocalisation to plasma membrane and recycling endosomes upon ITCH loss

Q11. AT2 stem cell self-renewal and proliferation in fetal lung organoids

Q12. FGF7-driven AT2 cell proliferation and surfactant processing balance

Q13. Expandable fetal-derived AT2 organoids maintaining identity over passaging

Q14. Alveolar type 1 cell differentiation from AT2 organoids via YAP activation

Q15. AT2 to AT1 lineage transition through Wnt withdrawal and LATS inhibition

Q16. AT1 cell fate markers AQP5 CAV1 AGER in differentiated fdAT2 organoids

Q17. CXCL chemokine expressing AT2 subpopulation in fetal lung organoids

Q18. Immune response gene expression in alveolar type 2 cells

Q19. Chemokine-mediated innate immune signaling in AT2 organoid subsets

Q20. Aberrant basal cell differentiation from AT2 cells in organoid culture

Q21. Hypoxia-induced airway differentiation of alveolar type 2 cells

Q22. Pulmonary neuroendocrine cell differentiation in AT2 organoids

Q23. Neuroendocrine progenitor cells co-expressing SFTPC and NE markers

Q24. Ciliated cell-like differentiation in fetal AT2 organoid culture

Q25. Intermediate transitional cell state between AT2 and differentiated lineages

Q26. Surfactant metabolism and lipid transport in fetal alveolar epithelium

Q27. Vesicle-mediated transport and lysosome localization in AT2 surfactant processing

Q28. Lipid storage membrane transport and vesicle cytoskeleton trafficking in AT2 cells

Q29. Wnt signaling pathway maintaining AT2 identity and inhibiting AT1 differentiation

Q30. SFTPC-I73T pathogenic variant causing interstitial lung disease and AT2 dysfunction

Q31. Toxic gain-of-function effect of misfolded surfactant protein C variants

Q32. Transcriptional maturity of fdAT2 organoids compared to adult AT2 and PSC-iAT2

Q33. Missing immune response MHC class II genes in fetal versus adult AT2 cells

Q34. CRISPRi-mediated depletion of ITCH and UBE2N in fdAT2 organoids

Q35. Reversible SFTPC mislocalization after CRISPRi recovery in AT2 organoids

Q36. ESCRT complex components HRS VPS28 required for SFTPC MVB entry

Q37. Endosomal recycling of SFTPC to plasma membrane upon ubiquitination failure

Q38. SUMOylation pathway components UBE2I UBA2 PIAS1 and SFTPC expression regulation

Q39. Fetal lung tip progenitor differentiation into mature AT2 cells

Q40. EpCAM positive tip epithelial cell isolation and AT2 organoid derivation

Q41. SFTPC C-terminal cleavage and proprotein processing in endosomal compartments

Q42. proSFTPC plasma membrane transit before endocytosis and maturation

Q43. Interstitial lung disease caused by SFTPC variants and AT2 cell dysfunction

Q44. Heritable pulmonary fibrosis from SFTPC mistrafficking and toxic accumulation

Q45. AT2 medium components dexamethasone cAMP IBMX DAPT for alveolar differentiation

Q46. fdAT2 organoid engraftment in mouse precision-cut lung slices and AT1 differentiation

Q47. NEDD4-2 HECT domain ligase role in SFTPC ubiquitination and maturation

Q48. Cell type heterogeneity and proportions across fdAT2 organoid lines

Q49. fdAT2 organoid stability over long-term passaging and cryopreservation

Q50. Genetic manipulation of fetal AT2 organoids using lentiviral CRISPRi system

S14.4.2 Expression queries

Q51. `SFTPC SFTPB SFTPA1 SFTPA2 NAPSA LAMP3`

Q52. `SFTPC SFTPB ABCA3 LAMP3 HOPX NKX2-1`

Q53. `NKX2-1 SLC34A2 LPCAT1 HOPX CEACAM6`

Q54. `SFTPC SFTPD SFTA3 CD36 CAV1 SLC34A2`

Q55. `SFTPA1 SFTPA2 SFTPB SFTPC SFTPD`

Q56. `ITCH UBE2N HRS VPS28 RABGEF1 EEA1`

Q57. `ITCH NEDD4 NEDD4L UBE2N UBE2I`

Q58. `EEA1 MICALL1 LAMP3 HRS VPS28`

Q59. `UBE2I UBA2 PIAS1 ITCH RABGEF1`

Q60. `ABCA3 LAMP3 NAPSA CKAP4 ZDHHC2 CTSH`

Q61. `ABCA3 SFTPB SFTPC LAMP3 P2RY2 LMCD1`

Q62. MKI67 PCNA TOP2A SFTPC NKX2-1

Q63. MKI67 PCNA CDK1 CCNB1 SFTPC

Q64. CXCL1 CXCL2 CXCL3 CCL2 SFTPC

Q65. CXCL1 CXCL3 CCL2 CCL4 CCL4L1

Q66. CXCL1 CXCL2 HLA-DPA1 HLA-DPB1 CCL2

Q67. HLA-DQB1 HLA-DMA HLA-DMB HLA-DRA HLA-DOA

Q68. HLA-DPA1 HLA-DPB1 HLA-DRA CD86 TNF

Q69. AQP5 CAV1 AGER HOPX

Q70. CAV1 AGER AQP5 PDPN

Q71. TP63 KRT5 KRT14 KRT15 SOX2

Q72. KRT5 KRT14 TP63 LAMB3 COL17A1

Q73. ASCL1 NEUROD1 GRP CHGA SYP CALCA

Q74. GRP ASCL1 SYT1 CHGA SYP

Q75. ASCL1 GRP SFTPC NKX2-1

Q76. FOXJ1 DNAH5 CAPS PIFO RSPH1

Q77. FOXJ1 DNAH5 DNAI1 RSPH1 CAPS

Q78. SOX2 SOX9 NKX2-1 SFTPC TP63

Q79. SOX2 NKX2-1 HOPX CAV1

Q80. CTNNB1 TCF7L2 AXIN2 WNT3A LGR5

Q81. SFTPC NKX2-1 HOPX SFTPB ABCA3 MKI67

Q82. NAPSA ABCA3 SFTA3 SFTPD LAMP3 HOPX

Q83. SFTPC ITCH EEA1 LAMP3 MICALL1 ABCA3

Q84. SFTPC NAPSA CTSH LAMP3 ITCH UBE2N

Q85. SFTPC CXCL1 CXCL2 NKX2-1 LAMP3

Q86. CDH1 TJP1 EPCAM SFTPC NKX2-1

Q87. ITCH HRS VPS28 UBE2N RABGEF1 PIAS1 UBE2I UBA2

Q88. ITCH NEDD4 NEDD4L HRS UBAP1 USP8

Q89. MKI67 TOP2A PCNA CDK1 CCNB1 CCNA2

Q90. SFTPC TP63 ASCL1 FOXJ1 NKX2-1

Q91. SFTPC SFTPB ASCL1 GRP TP63 KRT5

Q92. SFTPC CAV1 AGER AQP5 HOPX NKX2-1

Q93. LAMP3 ABCA3 SFTPB SFTPC NAPSA CD36

Q94. `CKAP4 ZDHHC2 SLC34A2 CTSH SFTPC`

Q95. `CXCL1 CXCL2 CXCL3 CCL2 CCL4 TNF`

Q96. `SOX9 NKX2-1 SFTPC SFTPB LAMP3`

Q97. `SFTPC NKX2-1 ASCL1 NEUROD1 GRP MKI67`

Q98. `SFTA3 SFTPD NAPSA NKX2-1 CKAP4 ZDHHC2 SLC34A2 CTSH SFTPA1 SFTPA2 SFTPC SFTPB`

Q99. `ITCH SFTPC LAMP3 ABCA3 UBE2N NAPSA`

Q100. `SFTPC CXCL1 MKI67 TP63 ASCL1 FOXJ1 SOX2 CAV1`

S14.5 D5: Healthy Breast Tissue Atlas (Bhat-Nakshatri *et al.* [5])

S14.5.1 Ontology queries

Q1. Luminal hormone sensing cells with estrogen receptor expression in the healthy breast

Q2. FOXA1 pioneer transcription factor activity in luminal hormone responsive breast epithelial cells

Q3. ERα–FOXA1–GATA3 transcription factor network in hormone responsive breast cells

Q4. Mature luminal cells with hormone receptor positive identity in breast tissue

Q5. Hormone sensing alpha versus beta cell states in breast epithelium

Q6. LHS cell-enriched fate factor DACH1 and PI3K pathway regulator INPP4B in breast

Q7. Lobular epithelial cells expressing APOD and immunoglobulin genes in breast

Q8. Luminal adaptive secretory precursor cells and progenitor identity in breast

Q9. ELF5 and EHF transcription factor expression in luminal progenitor breast cells

Q10. Alveolar progenitor cell state enriched in Indigenous American breast tissue

Q11. BRCA1 associated breast cancer originating from luminal progenitor cells

Q12. KIT receptor expression and chromatin accessibility in luminal progenitor cells

Q13. MFGE8 and SHANK2 expression in luminal progenitor cells of the breast

Q14. LASP basal–luminal intermediate progenitor cell identity in the breast

Q15. Basal-myoepithelial cells with TP63 and KRT14 expression in breast

Q16. Basal cell chromatin accessibility and TP63 binding site enrichment

Q17. Basal alpha and basal beta cell states in breast myoepithelium

Q18. SOX10 motif enrichment in basal-myoepithelial cells of the breast

Q19. KRT14 KRT17 expression in ductal epithelial and basal cells of breast tissue

Q20. Fibroblast heterogeneity and cell states in healthy breast stroma

Q21. Genetic ancestry-dependent variability in breast fibroblast cell states

Q22. Fibro-prematrix state enrichment in African ancestry breast tissue fibroblasts

Q23. PROCR ZEB1 PDGFRα multipotent stromal cells enriched in African ancestry breast

Q24. Myofibroblast and inflammatory fibroblast subtypes in breast cancer stroma

Q25. SFRP4 and Wnt pathway modulation in breast fibroblasts

Q26. Endothelial cell subtypes and vascular markers in breast tissue

Q27. Lymphatic endothelial cells expressing LYVE1 in breast stroma

Q28. ACKR1 stalk-like endothelial cell subtype in breast vasculature

Q29. Vascular endothelial cell heterogeneity in mammary gland microvasculature

Q30. Breast tissue angiogenesis and endothelial cell MECOM expression

Q31. T lymphocyte markers and immune cell identity in breast tissue

Q32. CD4 T cell IL7R expression and chromatin accessibility in breast

Q33. CD8 T cell GZMK cytotoxic activity and IFNG signaling in breast tissue

Q34. Tissue-resident memory T lymphocyte populations in healthy breast

Q35. Adaptive immune surveillance by T cells in mammary gland stroma

Q36. Macrophage identity and FCGR3A expression in breast tissue stroma

Q37. Macrophage subtypes and tissue-resident immune cells in healthy breast

Q38. Breast tissue-resident macrophage phagocytic function and complement expression

Q39. Myeloid lineage immune cells and monocyte-derived macrophages in mammary gland

Q40. Adipocyte subtypes and lipid metabolism in breast tissue

Q41. Adipocyte PLIN1 and FABP4 expression in healthy breast stroma

Q42. PLIN1 lipid droplet biology and adipocyte identity in mammary fat pad

Q43. Mammary gland adipose tissue and fatty acid binding protein expression

Q44. Epithelial cell hierarchy from basal to luminal hormone sensing in breast

Q45. CXCL12 chemokine expression in endothelial cells and fibroblasts of breast

Q46. VEGFA angiogenic signaling from luminal cells to endothelium in breast

Q47. IGF1 paracrine signaling from fibroblasts to luminal cells in breast stroma

Q48. Breast tissue microenvironment with stromal and immune cell interactions

Q49. Ancestry differences in breast tissue cellular composition and cancer risk

Q50. Gene expression differences between ductal and lobular epithelial cells of the breast

S14.5.2 Expression queries

Q51. FOXA1 ESR1 GATA3 ERBB4 ANKRD30A AFF3 TTC6

Q52. MYBPC1 THSD4 CTNND2 DACH1 INPP4B NEK10

Q53. ESR1 FOXA1 GATA3 ELOVL5 ANKRD30A

Q54. AFF3 TTC6 ERBB4 MYBPC1 THSD4

Q55. DACH1 NEK10 CTNND2 INPP4B ELOVL5

Q56. APOD IGHA1 IGKC ESR1 FOXA1 GATA3

Q57. DUSP1 DPM3 RPL36 IGHA1 IGKC APOD

Q58. ELF5 EHF KIT CCL28 KRT15 BARX2 NCALD

Q59. MFGE8 SHANK2 SORBS2 AGAP1 ELF5

Q60. KRT15 CCL28 KIT INPP4B ELF5

Q61. RBMS3 EHF BARX2 NCALD ELF5

Q62. ESR1 ELF5 EHF KIT CCL28

Q63. ELF5 KIT CCL28 EHF KRT15 BARX2

Q64. NCALD BARX2 SHANK2 SORBS2 MFGE8 ELF5

Q65. TP63 KRT14 KLHL29 FHOD3 SEMA5A

Q66. KLHL13 KLHL29 TP63 KRT14 PTPRT

Q67. TP63 KRT14 KRT17 FHOD3 ABLIM3

Q68. ST6GALNAC3 PTPRM SEMA5A KLHL29

Q69. KRT14 KRT17 TP63 KLHL29 KLHL13 FHOD3

Q70. LAMA2 SLIT2 RUNX1T1 COL1A1 COL3A1

Q71. COL3A1 POSTN COL1A1 IGF1 ADAM12

Q72. CFD MGST1 MFAP5 COL3A1 POSTN

Q73. PROCR ZEB1 PDGFRA COL1A1 LAMA2

Q74. SFRP4 COL1A1 POSTN LAMA2 SLIT2

Q75. COL1A1 PDPN CD34 CXCL12 LAMA2

Q76. MECOM LDB2 MMRN1 CXCL12 ACKR1

Q77. LYVE1 MECOM LDB2 MMRN1

Q78. ACKR1 CXCL12 MECOM LDB2

Q79. MECOM LDB2 MMRN1 LYVE1 ACKR1

Q80. CXCL12 MECOM LDB2 ACKR1 MMRN1

Q81. PTPRC SKAP1 ARHGAP15 THEMIS IL7R

Q82. `IL7R GZMK PTPRC SKAP1`

Q83. `IFNG GZMK IL7R THEMIS PTPRC`

Q84. `THEMIS ARHGAP15 SKAP1 PTPRC IL7R`

Q85. `PTPRC SKAP1 GZMK IFNG THEMIS ARHGAP15`

Q86. `FCGR3A ALCAM LYVE1 CD163`

Q87. `ALCAM FCGR3A LYVE1 CD14`

Q88. `FCGR3A ALCAM CD163 MERTK`

Q89. `ALCAM LYVE1 FCGR3A CD163 MARCO`

Q90. `PLIN1 FABP4 KIT ADIPOQ LEP`

Q91. `FABP4 PLIN1 ADIPOQ LEP LPL`

Q92. `PLIN1 FABP4 LPL PPARG ADIPOQ`

Q93. `FABP4 PLIN1 KIT ADIPOQ`

Q94. `FOXA1 ELF5 TP63 KRT14 GATA3 ESR1`

Q95. `GATA3 EHF ELF5 FOXA1 KRT15 KRT14 TP63`

Q96. `MECOM PTPRC FCGR3A PLIN1 LAMA2 TP63 FOXA1`

Q97. `CXCL12 LAMA2 MECOM LDB2 COL1A1`

Q98. `ESR1 FOXA1 ELF5 EHF KIT TP63 KRT14`

Q99. `PTPRC FCGR3A FABP4 PLIN1 MECOM`

Q100. `VEGFA LDB2 IGF1 LAMA2 FOXA1 ELF5`

S14.6 D6: First-Trimester Human Brain (Mannens *et al.* [6])

S14.6.1 Ontology queries

Q1. GABAergic inhibitory neuron differentiation in developing human midbrain

Q2. Midbrain GABAergic neuron OTX2 GATA2 TAL2 transcription factor expression

Q3. Cortical interneuron derived from medial ganglionic eminence LHX6 DLX2

Q4. Interneuron diversity parvalbumin somatostatin VIP subtypes developing cortex

Q5. TAL2 expressing midbrain GABAergic neurons linked to major depressive disorder

Q6. Lateral and caudal ganglionic eminence interneuron migration in telencephalon

Q7. Medial ganglionic eminence derived parvalbumin somatostatin interneuron

Q8. SOX14 expressing midbrain GABAergic neuron thalamic migration

Q9. Glutamatergic excitatory neuron in developing human telencephalon cortex

Q10. Telencephalic glutamatergic neuron LHX2 BHLHE22 cortical layer specification

Q11. Hindbrain glutamatergic neuron ATOH1 MEIS1 cerebellar granule cell

Q12. Deep layer cortical neuron FEZF2 BCL11B corticospinal projection

Q13. SATB2 expressing telencephalic excitatory neuron callosal projection

Q14. Upper layer cortical neuron CUX1 CUX2 RORB intracortical connectivity

Q15. EMX2 transcription factor dorsal telencephalon glutamatergic identity

Q16. Purkinje cell differentiation in developing cerebellum PTF1A ESRRB lineage

Q17. Purkinje neuron ESRRB oestrogen-related nuclear receptor cerebellum specific

Q18. Cerebellar Purkinje progenitor PTF1A ASCL1 NEUROG2 ventricular zone

Q19. TFAP2B LHX5 activation of ESRRB enhancer in Purkinje neuroblast

Q20. RORA FOXP2 EBF3 late Purkinje maturation gene regulatory network

Q21. Cerebellar granule neuron ATOH1 MEIS1 external granular layer

Q22. Radial glial cell neural stem cell SOX2 PAX6 NES in developing brain

Q23. Radial glia to glioblast transition NFI factor maturation NFIA NFIB NFIX

Q24. Neural progenitor cell proliferation and neurogenesis in ventricular zone

Q25. Loss of stemness and glial fate restriction by NFI transcription factors

Q26. Progenitor cell dividing in developing human brain VIM HES1 proliferating

Q27. Notch signaling DLL1 JAG1 NOTCH1 lateral inhibition neurogenesis

Q28. Glioblast astrocyte precursor GFAP S100B AQP4 BCAN TNC fetal brain

Q29. Astrocyte maturation and glial scar markers in developing brain

Q30. Oligodendrocyte precursor cell OLIG2 PDGFRA SOX10 specification

Q31. Oligodendrocyte differentiation MBP MOG PLP1 myelination fetal brain

Q32. Committed oligodendrocyte precursor SOX10 lineage commitment

Q33. Dopaminergic neuron midbrain TH NR4A2 substantia nigra ventral tegmental area

Q34. Serotonergic neuron raphe nucleus TPH2 SLC6A4 FEV brainstem

Q35. FOXA2 LMX1A floor plate derived dopaminergic neuron specification

Q36. Endothelial cell blood–brain barrier CLDN5 PECAM1 CDH5 fetal brain

Q37. Pericyte PDGFRB RGS5 FOXF2 cerebral vasculature developing brain

Q38. Vascular leptomeningeal cell FOXC1 meningeal fibroblast DCN COL1A1

Q39. Vascular smooth muscle cell ACTA2 MYH11 cerebral artery

Q40. Microglial cell CX3CR1 P2RY12 TMEM119 brain resident macrophage

Q41. Border-associated macrophage RUNX1 haematopoietic origin fetal brain

Q42. Immature T cell and leukocyte infiltration in developing fetal brain

Q43. Schwann cell MPZ CDH19 SOX10 neural crest derived myelinating peripheral glial

Q44. Sensory neuron dorsal root ganglion NTRK1 ISL1 peripheral nervous system

Q45. Glycinergic neuron SLC6A5 GLRA1 inhibitory spinal cord hindbrain

Q46. Neuroblast immature migrating neuron fetal cortex RBFOX3 NEFM

Q47. Major depressive disorder MDD midbrain GABAergic neuron NEGR1 LRFN5

Q48. Schizophrenia cortical interneuron medial ganglionic eminence SATB2

Q49. Attention deficit hyperactivity disorder ADHD cerebellar Purkinje

Q50. Autism spectrum disorder hindbrain neuroblast brainstem involvement

S14.6.2 Expression queries

Q51. `GAD1 GAD2 SLC32A1 DLX2 DLX5 LHX6`

Q52. `OTX2 GATA2 TAL2 SOX14 GAD2 SLC32A1`

Q53. `PVALB SST VIP LAMP5 SNCG ADARB2`

Q54. `DLX1 DLX2 DLX5 DLX6 MEIS2 LHX6`

Q55. `GAD1 GAD2 SLC32A1 TFAP2B OTX2`

Q56. `TAL2 SOX14 GAD2 OTX2 GATA2`

Q57. `SLC17A7 SLC17A6 SATB2 TBR1 FEZF2 BCL11B`

Q58. `EMX2 LHX2 BHLHE22 CUX1 CUX2 RORB`

Q59. `ATOH1 MEIS1 MEIS2 SLC17A6 RBFOX3`

Q60. `FEZF2 BCL11B TBR1 SATB2 SLC17A7`

Q61. `CUX1 CUX2 RORB LHX2 BHLHE22 EMX2`

Q62. `PTF1A ASCL1 NEUROG2 NHLH1 NHLH2 TFAP2B`

Q63. `ESRRB RORA PCP4 FOXP2 EBF3 LHX5`

Q64. `LHX5 LHX1 PAX2 TFAP2B DMBX1 NHLH2`

Q65. `ESRRB PCP4 RORA EBF1 EBF3 FOXP2 LHX1`

Q66. `SOX2 PAX6 NES VIM HES1 HES5 FABP7`

Q67. `NFIA NFIB NFIX SOX9 FABP7`

Q68. `SOX2 HES1 HES5 PAX6 NES VIM`

Q69. `NOTCH1 NOTCH2 DLL1 JAG1 HES1 HES5`

Q70. `GFAP S100B AQP4 ALDH1L1 BCAN TNC`

Q71. `OLIG1 OLIG2 SOX10 PDGFRA CSPG4`

Q72. `MBP MOG PLP1 MAG SOX10`

Q73. `OLIG2 SOX10 PDGFRA NKX2-2 OLIG1`

Q74. `TH DDC SLC6A3 SLC18A2 NR4A2 LMX1A FOXA2`

Q75. FOXA2 LMX1A NR4A2 TH DDC SLC18A2

Q76. TPH2 SLC6A4 FEV DDC SLC18A2

Q77. SLC6A5 GLRA1 SLC32A1 GAD1

Q78. RBFOX3 SNAP25 SYT1 NEFM NEFL TUBB3

Q79. NEFM NEFL MAP2 TUBB3 SYT1

Q80. CLDN5 PECAM1 CDH5 ERG FLT1 VWF

Q81. PDGFRB RGS5 ACTA2 MYH11 COL1A2

Q82. ACTA2 MYH11 PDGFRB TAGLN

Q83. DCN LUM COL1A1 COL1A2 FOXC1 COL3A1

Q84. FOXC1 FOXF2 DCN COL1A2 LUM

Q85. AIF1 CX3CR1 P2RY12 TMEM119 HEXB CSF1R

Q86. RUNX1 SPI1 CSF1R AIF1 CD68

Q87. AIF1 HEXB P2RY12 TMEM119 CX3CR1

Q88. CD3D CD3E CD3G PTPRC CD2

Q89. MPZ CDH19 SOX10 MBP PLP1

Q90. NTRK1 NTRK2 ISL1 PRPH SNAP25

Q91. RBFOX3 SLC17A6 GAD2 NEFM SNAP25

Q92. NEFM NEFL RBFOX3 TUBB3 DCX

Q93. NEGR1 BTN3A2 LRFN5 SCN8A RGS6 MYCN

Q94. OTX2 GATA2 MEIS2 PRDM10 MYCN

Q95. CTCF MECP2 YY1 RAD21 SMC3

Q96. SHH PTCH1 GLI1 GLI2 FOXA2 NKX2-1

Q97. WNT5A CTNNB1 LEF1 TCF7L2 AXIN2

Q98. BMP4 BMPR1A SMAD1 ID1 ID3

Q99. VEGFA KDR FLT1 PDGFB PDGFRB CLDN5

Q100. SOX2 PAX6 OLIG2 GFAP RBFOX3 GAD2 SLC17A7

S15 Example visualizations: cystic fibrosis dataset

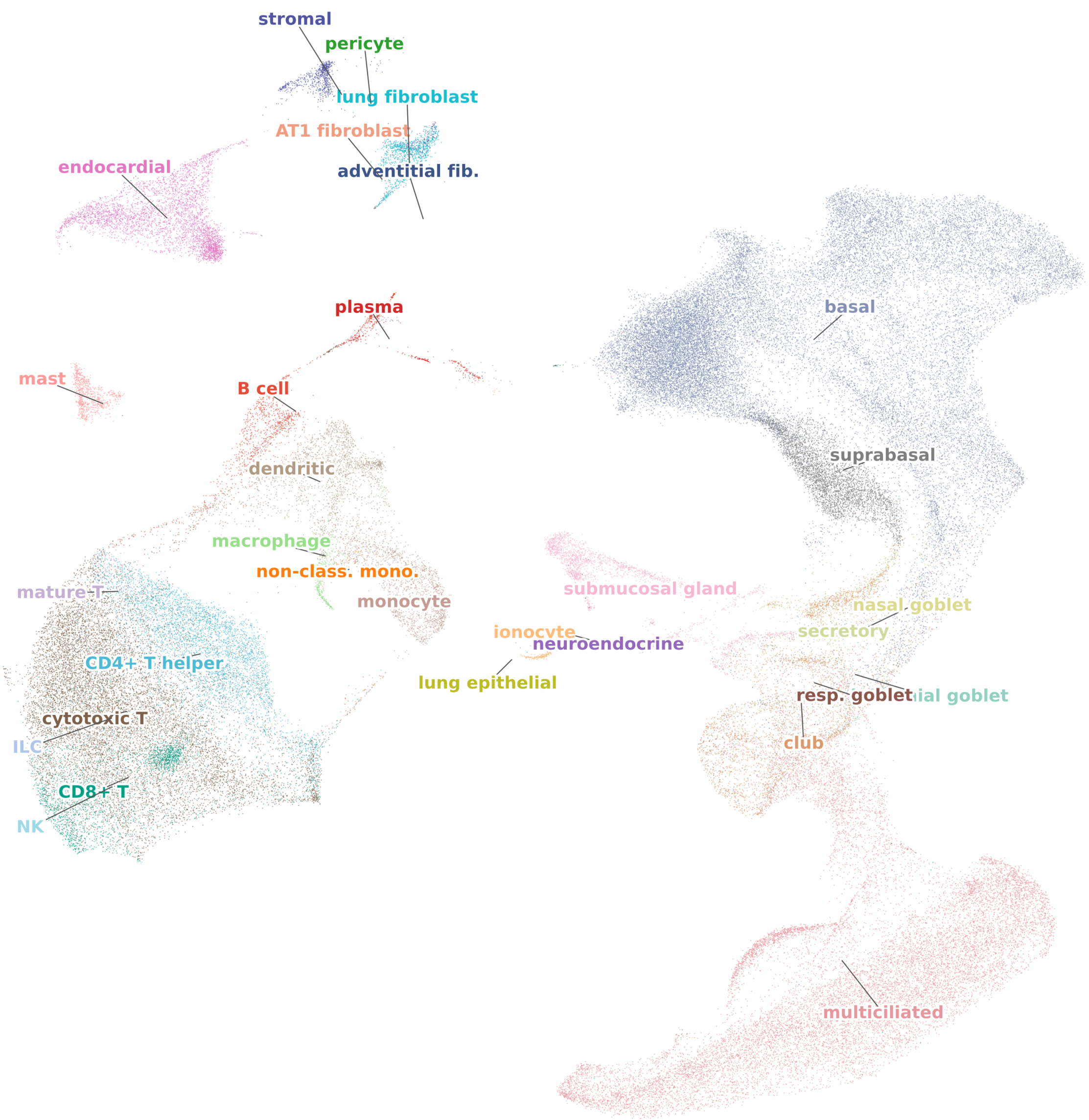

Figure S1: Cell-level UMAP of the cystic fibrosis airway dataset (D1) colored by Cell Ontology annotation. Approximately 96,000 cells are shown across 30 annotated cell types spanning immune (T cells, B cells, NK cells, macrophages, monocytes, dendritic cells, mast cells), epithelial (basal, suprabasal, multiciliated, secretory, goblet, club, ionocyte, neuroendocrine), and stromal (fibroblasts, pericytes, endocardial cells) compartments. Labels are placed at cluster centroids with iterative repulsion to minimize overlap.

Alt-text: A UMAP scatter plot of about 96,000 cystic fibrosis airway cells, grouped into 30 colour-coded clusters forming distinct immune, epithelial, and stromal regions.

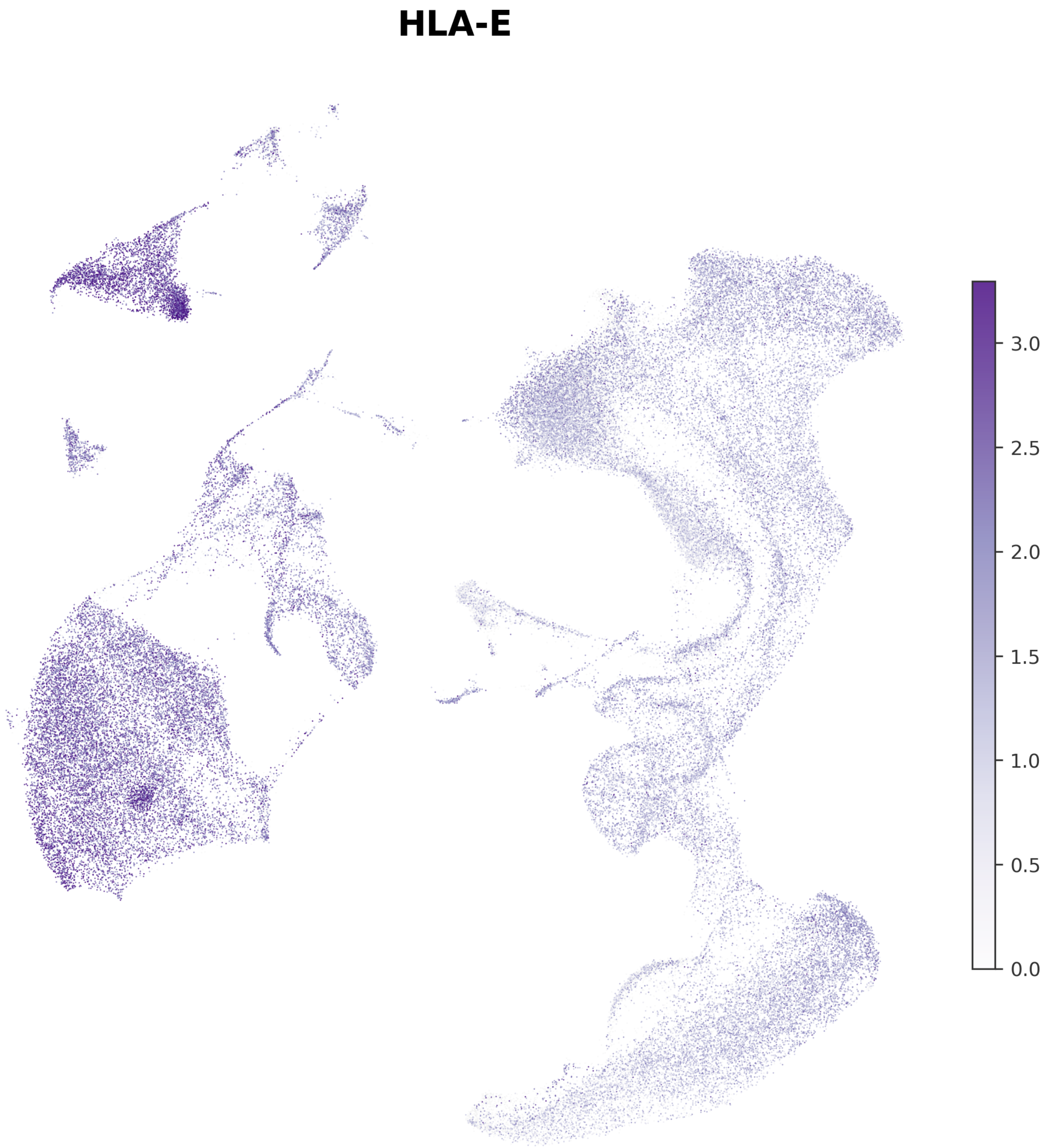

Figure S2: Expression of HLA-E projected onto the cell-level UMAP of the cystic fibrosis airway dataset (D1). Color intensity (purple gradient) indicates normalized expression level, with non-expressing cells shown in grey. HLA-E is most highly expressed in immune cell clusters, particularly $CD8^+$ T cells and NK cells, consistent with its role as a ligand for the NKG2A inhibitory receptor. Moderate expression is observed across epithelial populations including basal cells, supporting the HLA-E/NKG2A immune checkpoint axis identified by Berg *et al.* [1].

Alt-text: A UMAP feature plot where HLA-E expression appears as a purple gradient concentrated in the $CD8^+$ T cell and NK cell clusters, with lighter expression across epithelial populations.

References

[1] Marijn Berg, Lisette Krabbendam, Esmee K van der Ploeg, Menno van Nimwegen, Tjeerd van der Veer, Martin Banchero, Orestes A Carpaij, Remco Hoogenboezem, Maarten van den Berge, Eric Bindels, et al. Evidence for altered immune-structural cell crosstalk in cystic fibrosis revealed by single cell transcriptomics. *Journal of Cystic Fibrosis*, 2025.

[2] Wenbao Yu, Rumeysa Biyik-Sit, Yasin Uzun, Chia-Hui Chen, Anusha Thadi, Jonathan H Sussman, Minxing Pang, Chi-Yun Wu, Liron D Grossmann, Peng Gao, et al. Longitudinal single-cell multiomic atlas of high-risk neuroblastoma reveals chemotherapy-induced tumor microenvironment rewiring. *Nature Genetics*, 57(5):1142–1154, 2025.

[3] Mahnoor N Gondal, Marcin Cieslik, and Arul M Chinnaiyan. Integrated cancer cell-specific single-cell rna-seq datasets of immune checkpoint blockade-treated patients. *Scientific Data*, 12(1):139, 2025.

[4] Kyungtae Lim, Eimear N Rutherford, Livia Delpiano, Peng He, Weimin Lin, Dawei Sun, Dick JH Van den Boomen, James R Edgar, Jae Hak Bang, Alexander Predeus, et al. A novel human fetal lung-derived alveolar organoid model reveals mechanisms of surfactant protein c maturation relevant to interstitial lung disease. *The EMBO Journal*, 44(3):639, 2025.

[5] Poornima Bhat-Nakshatri, Hongyu Gao, Aditi S Khatpe, Adedeji K Adebayo, Patrick C McGuire, Cihat Erdogan, Duojiao Chen, Guanglong Jiang, Felicia New, Rana German, et al. Single-nucleus chromatin accessibility and transcriptomic map of breast tissues of women of diverse genetic ancestry. *Nature medicine*, 30(12):3482–3494, 2024.

[6] Camiel CA Mannens, Lijuan Hu, Peter Lönnerberg, Marijn Schipper, Caleb C Reagor, Xiaofei Li, Xiaoling He, Roger A Barker, Erik Sundström, Danielle Posthuma, et al. Chromatin accessibility during human first-trimester neurodevelopment. *Nature*, 647(8088):179–186, 2025.

[7] Haotian Cui, Chloe Wang, Hassaan Maan, Kuan Pang, Fengning Luo, Nan Duan, and Bo Wang. scgpt: toward building a foundation model for single-cell multi-omics using generative ai. *Nature methods*, 21(8):1470–1480, 2024.

[8] Nils Reimers and Iryna Gurevych. Sentence-bert: Sentence embeddings using siamese bert-networks. In *Proceedings of the 2019 conference on empirical methods in natural language processing and the 9th international joint conference on natural language processing (EMNLP-IJCNLP)*, pages 3982–3992, 2019.

[9] F Alexander Wolf, Philipp Angerer, and Fabian J Theis. Scanpy: large-scale single-cell gene expression data analysis. *Genome biology*, 19(1):15, 2018.

[10] Leland McInnes, John Healy, and James Melville. Umap: Uniform manifold approximation and projection for dimension reduction. *arXiv preprint arXiv:1802.03426*, 2018.

[11] Stephen Robertson and Hugo Zaragoza. *The probabilistic relevance framework: BM25 and beyond*, volume 4. Now Publishers Inc, 2009.

[12] Jinhyuk Lee, Wonjin Yoon, Sungdong Kim, Donghyeon Kim, Sunkyu Kim, Chan Ho So, and Jaewoo Kang. Biobert: a pre-trained biomedical language representation model for biomedical text mining. *Bioinformatics*, 36(4):1234–1240, 2020.

[13] Suoqin Jin, Maksim V Plikus, and Qing Nie. Cellchat for systematic analysis of cell–cell communication from single-cell transcriptomics. *Nature protocols*, 20(1):180–219, 2025.

[14] Mirjana Efremova, Miquel Vento-Tormo, Sarah A Teichmann, and Roser Vento-Tormo. Cellphonedb: inferring cell–cell communication from combined expression of multi-subunit ligand–receptor complexes. *Nature protocols*, 15(4):1484–1506, 2020.

[15] Robin Browaeys, Wouter Saelens, and Yvan Saeys. Nichenet: modeling intercellular communication by linking ligands to target genes. *Nature methods*, 17(2):159–162, 2020.

[16] Laurens van der Maaten and Geoffrey Hinton. Visualizing data using t-sne. *Journal of machine learning research*, 9(Nov):2579–2605, 2008.

[17] Aaron Grattafiori, Abhimanyu Dubey, Abhinav Jauhri, Abhinav Pandey, Abhishek Kadian, Ahmad Al-Dahle, Aiesha Letman, Akhil Mathur, Alan Schelten, Alex Vaughan, et al. The llama 3 herd of models. *arXiv preprint arXiv:2407.21783*, 2024.
